# The role of climate change and niche shifts in divergent range dynamics of a sister-species pair

**DOI:** 10.24072/pcjournal.248

**Published:** 2023-03-09

**Authors:** Jeremy Summers, Dieter Lukas, Corina J Logan, Nancy Chen

**Affiliations:** 1 University of Rochester, Rochester, NY, USA; 2 Max Planck Institute for Evolutionary Anthropology, Leipzig, Germany; 3 University of California Santa Barbara, Santa Barbara, CA, USA

## Abstract

Species ranges are set by limitations in factors including climate tolerances, habitat use, and dispersal abilities. Understanding the factors governing species range dynamics remains a challenge that is ever more important in our rapidly changing world. Species ranges can shift if environmental changes affect available habitat, or if the niche or habitat connectivity of a species changes. We tested how changes in habitat availability, niche, or habitat connectivity could contribute to divergent range dynamics in a sister-species pair. The great-tailed grackle (*Quiscalus mexicanus*) has expanded its range northward from Texas to Nebraska in the past 40 years, while its closest relative, the boattailed grackle (*Quiscalus major*), has remained tied to the coasts of the Atlantic Ocean and the Gulf of Mexico as well as the interior of Florida. We created species distribution and connectivity models trained on citizen science data from 1970–1979 and 2010–2019 to determine how the availability of habitat, the types of habitat occupied, and range-wide connectivity have changed for both species. We found that the two species occupy distinct habitats and that the great-tailed grackle has shifted to occupy a larger breadth of urban, arid environments farther from natural water sources. Meanwhile, the boattailed grackle has remained limited to warm, wet, coastal environments. We found no evidence that changes in habitat connectivity affected the ranges of either species. Overall, our results suggest that the great-tailed grackle has shifted its realized niche as part of its rapid range expansion, while the range dynamics of the boat-tailed grackle may be shaped more by climate change. The expansion in habitats occupied by the great-tailed grackle is consistent with observations that species with high behavioral flexibility can rapidly expand their geographic range by using human-altered habitat. This investigation identifies how opposite responses to anthropogenic change could drive divergent range dynamics, elucidating the factors that have and will continue to shape species ranges.

## Introduction

Species ranges determine the patterns of biodiversity across the world, shaping the environments different species encounter and the other species they can interact with (Gaston, 1996, 2003; Holt, 2003). We are still determining how abiotic and biotic factors limit species ranges (Buckley et al., 2018; Paquette and Hargreaves, 2021; Sirén and Morelli, 2020) and to what degree a species is able to expand to new habitats (Holt, 2003; Ralston et al., 2016). Within the limits that determine species ranges, many animal species today are experiencing massive declines due to loss of habitat (IUCN, 2022). These declines have been linked to limitations in the ability of many species to change their realized niche, the range of habitats that these species occupy, despite movement to new geographic areas or environmental change (Holt and Gaines, 1992; Liu, Wolter, et al., 2020; Wiens et al., 2010). The realized niche of a species is the result of environmental limitations due to physiology and behavior, geographic limitations due to dispersal, and ecological limitations due to interspecific interactions. Together, these three limitations determine species ranges (Soberón and Nakamura, 2009). However, some species can change their realized niche through occupying novel environmental conditions, a process referred to as a niche shift (Broennimann, Treier, et al., 2007; Guisan et al., 2014; Hill et al., 2017; Sherpa et al., 2019), potentially allowing them to expand their ranges while other species cannot (Holt, 2003; Holt and Gaines, 1992; Wiens et al., 2010). The factors that allow some species to shift their niche have remained difficult to identify (Wiens et al., 2010).

A species expanding into new areas is assumed to have overcome some of the trade-offs or limitations that shape a species’ realized niche. Niche shifts can occur via physiological or behavioral changes, as well as interactions between these factors (Wiens et al., 2010). Physiological changes reflect evolutionary changes in the phenotypes of individuals, such as changes in body size or metabolic processes, through which individuals of a species can occupy different niches (Buckley et al., 2018). Such physiological changes often occur over longer time spans (Swanson and Garland, 2009), suggesting that fast expansions into new niches are presumably facilitated by already existing plasticity in physiological tolerances. One potential cause of niche shifts over shorter time spans is behavioral flexibility, the ability to change behavior when circumstances change [see Mikhalevich et al., 2017 for theoretical background on our flexibility definition (Chow et al., 2016; Griffin and Guez, 2014; e.g., Lefebvre et al., 1997; Sol, Duncan, et al., 2005; Sol and Lefebvre, 2000; Sol, Székely, et al., 2007; Sol, Timmermans, et al., 2002). This idea predicts that flexibility, exploration, and innovation facilitate the expansion of individuals into completely new areas and that the role of these characteristics diminishes after some number of generations (Wright, Eberhard, et al., 2010). Experimental studies have shown that latent abilities are primarily expressed in a time of need (Auersperg et al., 2012; Bird and Emery, 2009; Laumer et al., 2018; Manrique and Call, 2011; e.g., Taylor et al., 2007). Therefore, we do not expect the founding individuals who initially dispersed out of their original range to have unique behavioral characteristics that are passed on to their offspring. Instead, the actual act of continuing a range expansion likely relies on flexibility, exploration, innovation, and persistence, and thus these behaviors should be expressed more on the edge of the expansion range where there have not been many generations to accumulate relevant knowledge about the environment (Magory Cohen et al., 2020; Nicolaus et al., 2022; Sol, Stirling, et al., 2005; Wright, Eberhard, et al., 2010). There is also evidence that some species can behaviorally shift their niche in response to anthropogenic climate change or that they can expand their range by using human altered environments (Wolff et al., 2020; Wong and Candolin, 2015). Human-modified environments are increasing (Goldewijk, 2001; e.g., Liu, Wolter, et al., 2020; Wu, Jenerette, et al., 2011), and species associated with these habitats show differences in their behavior (Chejanovski et al., 2017; e.g., Ciani, 1986; Federspiel et al., 2017).

However, range dynamics are also influenced by factors beyond changes in the realized niche: environmental change leading to a recent increase in the amount of available habitat representing the current niche can facilitate a geographic range expansion (Hanski and Gilpin, 1991; Wiens, 1997), and change in habitat connectivity can alter species range limits (Holt, 2003; Platts et al., 2019). A species may not need to be behaviorally flexible to move into new areas if it can continue to use the same habitats within its expanded range. For example, a species may expand its range because changes in climate have caused more geographic areas to fall within its niche or if previously isolated habitat patches become connected. Thus, it is important to identify how changes in the availability of habitats, the usage of different habitats, and habitat connectivity contribute to range shifts to understand whether niche shifts are truly happening and to identify potential causes of range shifts.

**Figure 1 –:**
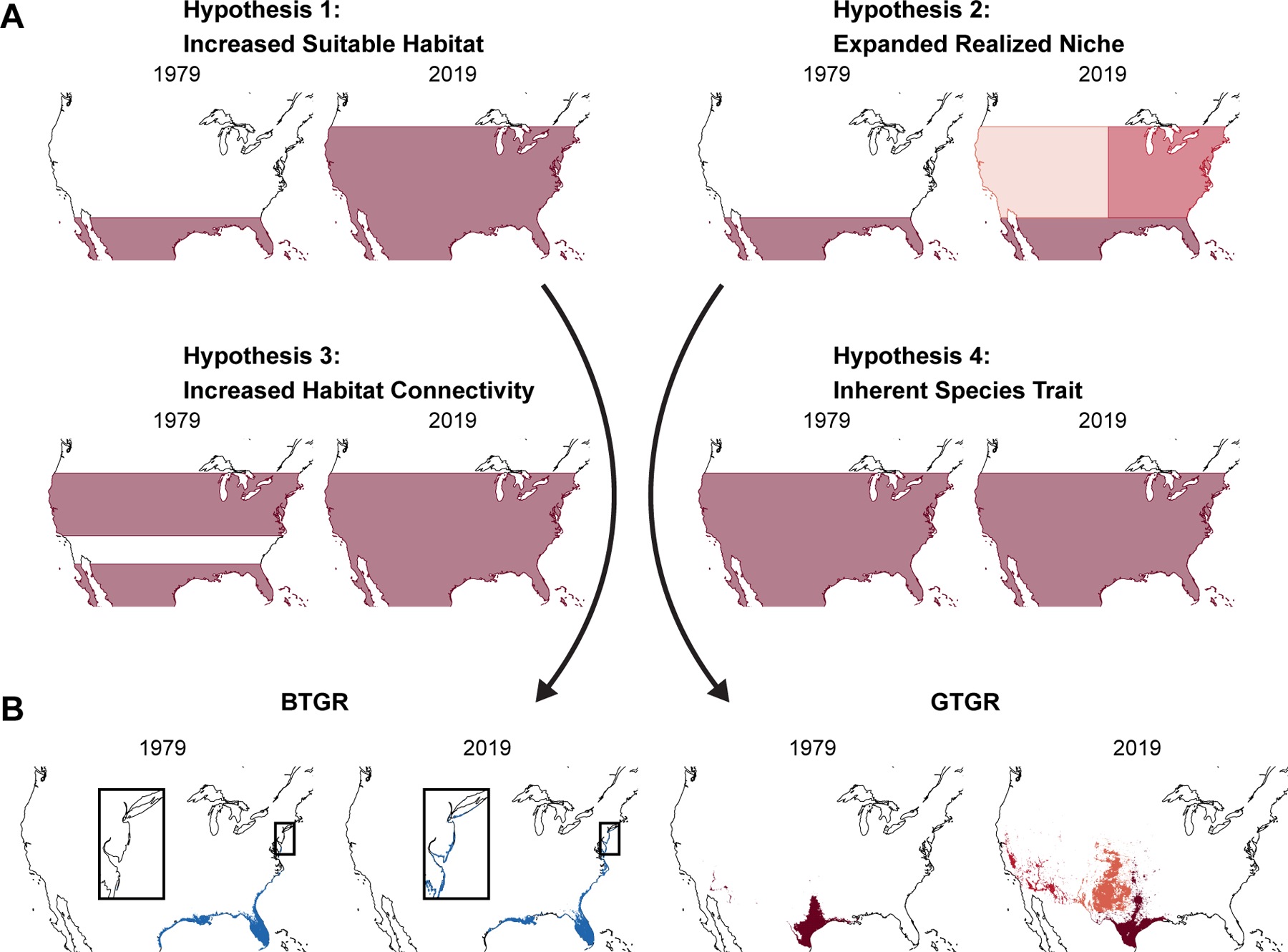
Comparison between the predicted patterns depending on the forces that facilitated range expansion and habitat suitability predicted by the species distribution models (SDMs) for the great-tailed grackle (GTGR) and boat-tailed grackle (BTGR). (A) The pairs of plots display the predictions for the historic and current models if increased suitable habitat (Hypothesis 1), expanded realized niche (Hypothesis 2), increased habitat connectivity (Hypothesis 3), or other inherent species trait(s) (Hypothesis 4) drove range expansion. (B) The suitable habitat predictions for the historic and current models based on environmental data from 1979 and 2019. We used the maximum-sensitivity-specificity thresholds for each model (great-tailed grackle current: 0.4440, boat-tailed grackle current: 0.4780, great-tailed grackle historic: 0.4635, boat-tailed grackle historic: 0.3935) to assign habitat as suitable. The different colors in the great-tailed grackle map indicate that some environmental conditions within its 2019 expanded range were not found in its 1979 range. The arrows connect the species ranges to the most supported predicted range dynamics.

Here we investigated the drivers of different range dynamics in two closely related grackle species, the great-tailed grackle (*Quiscalus mexicanus*) and boat-tailed grackle (*Quiscalus major*). These species offer an opportunity for simultaneous investigation of the roles of behavior and increased habitat availability in a rapidly increasing geographic range expansion. The great-tailed grackle has rapidly expanded its range northward over the course of the 20th century (Post et al., 1996; Wehtje, 2003), moving its northern range edge from Southern Texas to Nebraska (Fig 1B). In contrast, the boat-tailed grackle range has remained largely the same, with only minor changes to the northern edge of its range (Wehtje, 2003), despite both species having similar foraging habits and successfully using human-altered environments (Johnson and Peer, 2020; Post et al., 1996; Selander and Giller, 1961). The great-tailed grackle is highly behaviorally flexible (Logan, 2016a,b), similar to other species that successfully use human-altered environments (Wong and Candolin, 2015), but the behavioral flexibility of the boat-tailed grackle has not yet been assessed. Detailed reports on the breeding ecology of these two species indicate that range expansion in the boat-tailed grackle but not the great-tailed grackle may be constrained by the availability of suitable nesting sites (Selander and Giller, 1961; Wehtje, 2003). Boat-tailed grackles may be limited by the need for coastal marshes or isolated groves near water for nesting sites (Post et al., 1996), while great-tailed grackles can nest in agricultural lands, marshes, and urban areas with vegetation and surface water (Johnson and Peer, 2020). Great-tailed grackles inhabit a wide variety of habitats (but not forests) at a variety of elevations (0–2134m), while remaining near water bodies. Boat-tailed grackles exist mainly in coastal areas (Selander and Giller, 1961). There is also evidence that great-tailed grackles have preferred different habitats over time and across their range. Ornithologists have recorded great-tailed grackles breeding primarily in natural and human-made wetlands, while those within the recently expanded range readily breed in urban parks (Wehtje, 2003). However, this apparent difference in niche has yet to be rigorously quantified.

The range expansion in the great-tailed grackle and range stability in the boat-tailed grackle could be due to differences in realized niche change between these two closely related species. We characterized the historic (1970–1979) and current (2010–2019) realized niches of the great-tailed grackle and the boat-tailed grackle using species distribution models (SDMs) to test three hypotheses on the causes of range expansion in the great-tailed grackle and range stability in the boat-tailed grackle (Fig 1A). **Hypothesis 1: change in habitat availability:** The great-tailed grackle and the boat-tailed grackle use different habitats, and the suitable habitat of the great-tailed grackle, but not that of the boat-tailed grackle, has increased northward over the past few decades. We define habitat suitability in this paper as the predicted habitat suitability for occupancy by the focal species, habitat that is within the limits of tolerability of the climate and environmental factors as determined by the areas occupied by individuals of the species at a given time. Support for this hypothesis would indicate that the availability of habitat due to environmental change, not inherent species differences, explains why the great-tailed grackle has rapidly expanded its range while the boat-tailed grackle has not. **Hypothesis 2: change in realized niche:** Over the past few decades, the great-tailed grackle has expanded its realized niche, whereas the boat-tailed grackle continues to use the same limited habitat types. In other words, a niche shift, possibly due to changes in behavioral traits, facilitated the geographic range expansion of the great-tailed grackle. **Hypothesis 3: changes in habitat connectivity:** Species distribution models generally do not account for additional factors such as dispersal limitations due to landscape heterogeneity when estimating suitable habitat. Therefore, we conducted a separate analysis to examine possible changes in connected habitat due to environmental change. Support for this hypothesis would indicate that environmental change has facilitated the range expansion of the great-tailed grackle. **Hypothesis 4: inherent species trait(s):** Other species traits, such as demographic dynamics or dispersal physiology, limited the historic species range, resulting in no apparent environmental difference between the newly occupied and historically occupied ranges. Given this hypothesis, there are no changes in habitat availability, but both species have suitable but unoccupied habitat available to them. Only the great-tailed grackle is able to occupy additional habitat due to changes in the other traits or conditions that previously limited the species range, with the ongoing expansion reflecting the time-lag to reach new areas. This outcome would be consistent with the hypothesis that the original behavior of the great-tailed grackle, determined by inherent species traits, was already well adapted to facilitate a range expansion while the behavior of the boat-tailed grackle restricts it to its current range.

**Figure 2 –:**
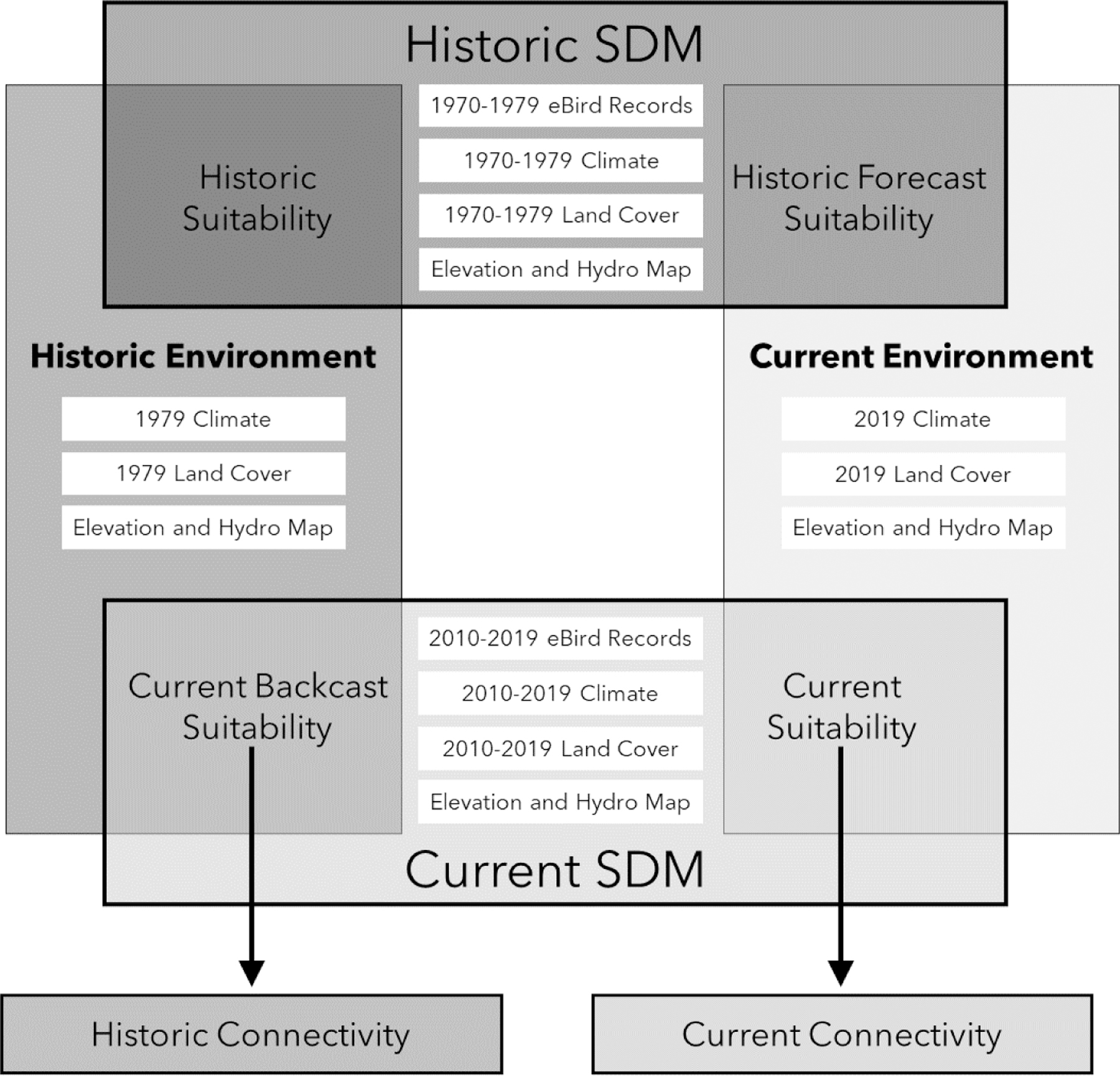
Overview of modeling approach and steps. The white boxes list the data used to generate the species distribution models (SDMs) and environments used for predicting habitat suitability. The overlap between shaded boxes indicates that a habitat suitability prediction was created using the overlapping species distribution model and environmental predictors. The arrows indicate the habitat suitability predictions used to create the connectivity models (see Methods for a detailed description of data sources and steps).

We used ecological niche modeling to examine temporal habitat changes over these past four decades using observation data for both grackle species from existing citizen science databases. We determined the change in habitat availability using predictions produced by both our current and historic models for each species based on environmental data from 1979 and 2019 (Fig 2, Analysis 1). We also tested the ability of our current and historic models to predict species presence and absence using data from the opposite time period to validate the predicted changes in suitable habitat (Regos, Imbeau, et al., 2018; Torres et al., 2015; Yates et al., 2018) (Analysis 1). Together, the components of Analysis 1 address Hypothesis 1 that environmental change could have led to the range dynamics seen in both species. Then, we compared how the importance and effect of environmental predictors (Analysis 2) and occupied environments changed between our current and historic models (Analysis 3). Analyses 2 and 3 both address Hypothesis 2, that changes in the types of habitat occupied could have led to the observed range dynamics. Finally, we used a circuit theory-based connectivity model to test for changes in habitat connectivity between 1979 and 2019 (Analysis 4), which addresses Hypothesis 3, that changes in habitat connectivity caused by environmental change could have led to the observed range dynamics. Finally, the overall power of our analyses to predict the range dynamics of the great-tailed grackle addresses Hypothesis 4. If inherent species traits are a main component of the observed range dynamics, our species distribution and connectivity models should not be able to fully differentiate the realized niche and geographic areas occupied by the great-tailed grackle over time, as these models do not account for those traits. A range increase even though changes in the environment, realized niche of the great-tailed grackle, and landscape connectivity have not increased the geographic areas of suitable and accessible habitat over time would indicate that great-tailed grackles already had the inherent ability to occupy the newly inhabited areas. In combination, our analyses allowed us to investigate whether the range of the great-tailed grackle, but not the boat-tailed grackle, might have increased due to an increase in habitat availability, expansion of the realized niche of the great-tailed grackle, or changes in habitat connectivity.

## Methods

This article is the first of three articles that will be produced from a preregistration (http://corinalogan.com/Preregistrations/gxpopbehaviorhabitat.html) that passed pre-study peer review at Peer Community in Ecology in 2020. The hypotheses, predictions, and methods in this manuscript come from the preregistration, and we detail all changes to the methods below.

### Preregistered Analysis Plan

#### Response Variable:

Presence/absence of great-tailed grackles and boat-tailed grackles

### Explanatory Variables

1. **Land cover** (e.g., forest, urban, arable land, pastureland, wetlands, marine coastal, grassland, mangrove) - we chose these land cover types because they represent the habitat types in which both species exist, as well as habitat types (e.g., forest) they are not expected to exist in (Selander and Giller, 1961). If the suitable and unsuitable habitat of the great-tailed grackle agrees with these expectations, it is possible that large forested areas are barriers for the range expansion of one or both species. We planned to download global land cover type data from MODIS (16 terrestrial habitat types) and/or the IUCN habitat classification (47 terrestrial habitat types). The IUCN has assigned habitat classifications for the great-tailed grackle and the boat-tailed grackle; however, these classifications appear to be out of date, and we updated them for the purposes of this project.
  - **Further details:** We limited our study extent to the contiguous United States, which should not affect our investigation of distribution changes because the entire range of the boat-tailed grackle and the northern expanding edge of the great-tailed grackle range are both within the contiguous United States. We verified this assumption by comparing species distribution models using 2010–2019 observations and MODIS land cover data with and without the limited spatial extent. Restricting the training data to the contiguous United States caused no drop in the AUC when predicting habitat suitability within the US relative to the unrestricted model.
  - **Deviations from the preregistered plan:** We used the National Land Cover Database (NLCD) (Homer et al., 2015) and historical land cover modeling data from Sohl et al., 2016 instead of MODIS for our land cover dataset because the former datasets have a greater temporal range. MODIS data exists for a continuous period of 2001-present, and could only be extended to 1993 using compatible data from the Global Land Cover Characterization (GLCC) land cover dataset. Using MODIS data would require limiting the temporal range of our study to 1993-present, yet the most rapid period of the great-tailed grackle expansion occurred from 1967–1977 (Wehtje, 2003). We initially proposed to use data from 1968–1970 for our historical model, and data from 2018 for our present-day model. Instead, we used land cover projections from Sohl et al., 2016 for our historical land cover data (1970–1979) and the NLCD (2011, 2013, 2016; and 2019) for our modern land cover data, which allowed us to model species distributions closer to our proposed temporal range. Both datasets use a modified version of the Anderson Land Classification System (Anderson and Hardy, 1971), share the same geographic extent, and are high resolution (250m and 30m, respectively). The land cover classification system includes classes for forests, urban areas, pasture and crop lands, wetlands, and grasslands.
2. **Elevation** - Selander and Giller, 1961 notes the elevation range for the great-tailed grackle (0–2134m), but not the boat-tailed grackle, therefore establishing that the current elevation ranges for both species may allow us to determine whether and which mountain ranges present range expansion challenges. We obtained elevation data from the Global Multi-resolution Terrain Elevation Data 2010 (GMTED2010; Danielson and Gesch, 2011) available through USGS.
3. **Climate** (e.g., daily/annual temperature range) - the great-tailed grackle was originally from the tropics (Wehtje, 2003), which generally have a narrow daily and annual climate range, and now exists in temperate regions, which have much larger climate ranges. Ac-cordingly, the daily/annual temperature range could allow us to determine the role of potential climatic limits in explaining ranges and range changes for both species. If there are limits, climate conditions could inform the difference between the range expansion rates of the two species. We considered the 19 bioclimatic variables from WorldClim.
  - **Further details:** We converted monthly climate data for each time period from World-Clim (Fick and Hijmans, 2017) into the set of 19 climate variables included in the BioClim dataset using the *biovars* function from the dismo package in R (Hijmans, Phillips, et al., 2022). We tested the 19 BioClim variables across the ranges of both species for collinearity using the *vifcor* function from the usdm package in R (Naimi et al., 2014) with a correlation threshold of 0.7. For highly correlated variables, we excluded the variable with the greater variable inflation factor. Our final dataset included 7 climate variables: mean diurnal temperature range, maximum temperature of the warmest month, mean temperature of the wettest quarter, precipitation of the wettest month, precipitation of the driest month, and precipitation of the coldest quarter.
4. **Presence/absence of water in the cell for each point** - both species are considered to be highly associated with water (e.g., Selander and Giller, 1961, therefore we identified how far from water each species can exist to determine whether it is a limiting factor in the range expansion of one or both species. We had planned to use data from USGS National Hydrography.
  - **Further details:** We separated the coastlines and bodies of freshwater due to the associations the boat-tailed grackle has with salt water (Post et al., 1996) and the great-tailed grackle has with freshwater (Selander and Giller, 1961).
  - **Deviations from the preregistered plan:** We used the river, lake, and coastline shape-files from the Natural Earth database (http://www.naturalearthdata.com/) as the basis for water bodies instead of the USGS National Hydrography database. The USGS National Hydrography database does not differentiate between minor and major bodies of water, resulting in near-complete coverage of the contiguous US map with bodies of water. The Natural Earth database incorporates data on rivers and lakes from the North American Environmental Atlas at a 1:10 million scale. The lower resolution data allowed for the computation of distances between the more than 1 million sample points and all water bodies. Natural Earth shapefiles have also been used in other SDMs to calculate distances to water bodies (Mi et al., 2017).
5. **Connectivity:** We planned to use connectivity as the distance between points on the northern edge of the range to the nearest uninhabited suitable habitat patch to the north in 1970 compared with the same patches in ~2018. We identified the northern edge of the distribution based on reports on eBird.org from 1968–1970, which resulted in recordings of great-tailed grackles in 48 patches and recordings of boat-tailed grackles in 30 patches. For these patches, we calculated the connectivity (the least cost path) to the nearest uninhabited suitable habitat patch in 1970 and again in ~2018. Given that great-tailed grackles are not found in forests or beyond certain elevations (Selander and Giller, 1961), large forests and high elevation geographic features could block or slow the expansion of one or both species into these areas and their surroundings. For each point, we planned to calculate the least cost path between it and the nearest location with grackle presence using the leastcostpath R package (Lewis, 2022). This approach would allow us to determine the costs involved in a grackle’s decision to fly around or over a mountain range/forest. We would define the forest and mountain ranges from the land cover and/or elevation maps.
  - **Deviations from the preregistered plan:** We did not include connectivity as an explanatory variable within our SDMs because we used a method for calculating connectivity that was dependent on the output of our SDMs. We quantified changes in connectivity using Circuitscape version 4.0.5 (Anantharaman et al., 2020), a method that uses electrical circuit theory, treating a landscape as an electrical circuit with different landscape features offering different levels of resistance. We created our resistance surfaces using the results of our SDMs, which is a common practice when experimental data on species movement through a landscape is not available (Beier et al., 2011; Justen et al., 2021; Miranda et al., 2021). See the Analysis 4 section below for more details on our connectivity models.

### Species Distribution Models

One model, including all explanatory variables, was run for the great-tailed grackle and a separate model was run for the boat-tailed grackle. We planned to use the program MaxEnt (Phillips and Dudík, 2008) to create the species distribution models. MaxEnt is a maximum entropy based software that compares environments between species presence and a set of background points to estimate habitat suitability (Phillips and Dudík, 2008). For the explanatory variables, MaxEnt produces a continuous prediction of habitat suitability for each grid cell (0 is least suitable and 1 is most suitable). We planned to use MaxEnt followed by jackknifing procedures to evaluate the relative contribution/importance of different environmental variables to the probability of species occurrence. We planned to optimize the model by trying different regularization coefficient values, which controls how much additional terms are penalized (Maxent’s way of protecting against overfitting), and choosing the value that maximizes model fit. Most MaxEnt papers use cross-validation and the area under the curve (AUC) to evaluate model performance, and we planned to do the same.

For all models we fit, we selected one presence and one absence from a 2.5 km hexagonal grid per week to geographically subsample the data and reduce imbalance in observation effort. We then separated the subsampled checklists into a set to train our model (80% of checklists) and a set for model validation (20% of checklists). We used a balanced random forest approach, in which absence points are selected at an equal frequency as presence points, thus addressing the imbalance in the ratio of presence and absence points (Strimas-Mackey, Hochachka, et al., 2020). Random forests are machine learning algorithms that generate a large number of classification trees based on different subsets of the given data (Evans et al., 2011). Once all trees are generated, the average result is taken and used as the final classification method, which determines which environmental factors differentiate species presences from species absences. We accounted for stochasticity in the geographic subsampling, dataset separation, and balanced random forest processes by repeating model creation 10 times independently for each time period and species. We used the ranger package in R to create each model (Wright and Ziegler, 2017).

We predicted habitat suitability across the contiguous United States using environmental data from 1979 and 2019. We produced three types of predictions (contemporary predictions, forecasts, and backcasts) depending on whether the time period of the SDM matched the time period of the environmental data (Fig 2). When the time periods matched, we produced contemporary predictions (e.g., predictions using the historic great-tailed grackle model with the 1979 environmental data). The predictions we made using the historic models and the 2019 environmental data were forecast predictions, and the predictions we made using the current model and the 1979 environmental data were backcast predictions. To standardize the predicted suitabilities, we set all effort covariates to the same values within the models of each species. We set the day of the year to April 1st, the observation time to maximize the encounter rate for each species (5 AM for the boat-tailed grackle and 6 AM for the great-tailed grackle, based on most common observation times), observation duration to one hour, distance traveled to one km, and the number of observers to one. We present the average habitat suitability predicted by the 10 replicates of each model.

- **Deviations from the preregistered plan:** We used a random forest model to estimate habitat suitability in place of Maxent due to the advantages offered by using presence-absence data instead of presence-background data. Presence-background data can only determine the habitat suitability of points relative to the background environment (Guillera-Arroita, Lahoz-Monfort, and Elith, 2014), thus the results of presence-background models such as Maxent cannot be compared between different environments due to the difference in backgrounds. This limitation of presence-background models makes them a poor fit for comparing range shifts over long periods of time (Sofaer et al., 2018). In contrast, presence-absence data allows relative likelihood to be proportional to the probability of occurrence so long as the sampling process is included within the model through effort covariates (Guillera-Arroita, Lahoz-Monfort, Elith, et al., 2015). Random forest models incorporate absence points and are similarly robust to limited sample sizes and against overfitting as are Maxent models (Elith and Graham, 2009; Evans et al., 2011; Mi et al., 2017; Norberg et al., 2019). Random forest models have also been used to fit species distribution models based on citizen science data (Robinson et al., 2020), including in the best practices for eBird data (Strimas-Mackey, Hochachka, et al., 2020). Johnston et al., 2021 directly compared Maxent and random forest models using eBird data and found that the random forest model that included effort covariates performed the best in terms of the AUC and Cohen’s Kappa. Cohen’s Kappa is a chance-corrected measurement of agreement between groups made by a classification system and a set of samples classified into real values (Titus and Mosher, 1984). We fit species distribution models based on the 2010–2019 data for the great-tailed grackle and the boat-tailed grackle using both random forest and Maxent and found that the random forest model outperformed the Maxent model based on AUC and kappa for both species. The data preparation methods have remained the same, and the models still output a continuous habitat suitability metric between 0 and 1 for each grid cell.

### Analysis instructions

1. Download and preprocess eBird data. Conduct spatial filtering to account for sampling bias
2. Clean the species occurrence data: remove any uncertain records or geographic outliers
3. Import climactic variables from WorldClim and landscape data from MODIS and crop to region of interest
4. Match environmental data to grackle occurrence records
5. Fit models with maxent to get predicted distributions and estimate importance/contribution of each environmental variable

We referred to Strimas-Mackey, Hochachka, et al., 2020 when extracting data on grackle presence in a region from eBird.org. We planned to gather environmental data from databases, including a database that maps global urban change from 1985–2015 to a high (30 m) resolution (Liu, Wolter, et al., 2020). We used a variety of R packages, including auk (Strimas-Mackey, Miller, et al., 2022), dismo (Hijmans, Phillips, et al., 2022), raster (Hijmans, Etten, et al., 2023), maptools (Bivand, Lewin-Koh, et al., 2022), tidyverse (Wickham et al., 2019), rgdal (Bivand, Keitt, et al., 2023), rJava (Urbanek, 2021), and elevatr (Hollister et al., 2022).

We used the R package auk (Strimas-Mackey, Miller, et al., 2022) to download and process occurrence records for both the great-tailed grackle and the boat-tailed grackle from the citizen science project eBird (Sullivan et al., 2014), matching our preregistered analysis plan. We included only complete checklists to allow us to infer non-detections (Johnston et al., 2021). We filtered the selected checklists to only include those less than 5 hours long, less than 5 km in length, and with fewer than 10 observers, in accordance with recommendations from (Strimas-Mackey, Hochachka, et al., 2020). We also excluded presence points outside the current known range for either species (Johnson and Peer, 2020; Post et al., 1996). We kept all checklists within 600 km of the remaining presence points to restrict our datasets to areas near the species ranges while including a wide area of environmental conditions. We also included information on the year of observation, day of the year, time of observation, distance traveled, observation duration, and number of observers as effort covariates for use in our SDMs. In total, we included 8,163 historic and 8,606,111 current great-tailed grackle checklists (with 502 and 519,082 great-tailed grackle observations, respectively) and 6,940 historic and 7,211,101 current boat-tailed grackle checklists (with 467 and 304,028 boat-tailed grackle observations, respectively). All species observation locations can be found in Supplementary Figure S1.

- **Deviations from preregistered plan:** For our historic models, we used checklists from 1970–1979, and for the current models we used checklists from 2010–2019 (EBD_relJan-2021, 2021) instead of 1960 and 2018, respectively. The temporal ranges for our dataset were selected for both sufficient sample size and overlap with the period of maximum great-tailed grackle range expansion (Wehtje, 2003). To determine the minimum number of samples needed to make our present and historical models comparable, we created species distribution models using subsamples of the 2010–2019 eBird dataset with different numbers of positive observations. We found that retaining 300 observations allowed our models to have a ∆AUC of less than 0.1. Using this limit, we set the temporal range for our historical model to 1970–1979 because this range had ≥ 300 observations of both species and captures the most rapid period of great-tailed grackle range expansion. We also limited our spatial extent to the contiguous United States to ensure consistent coverage of historic and current environmental data.

#### Analysis 1: habitat availability:

Has the available habitat for both species increased over time? We fit species distribution models for both species in 1970 and in 2018 and determined for each variable, the range in which grackles were present (we define this area as the habitat suitability for each species). We then planned to take these variables and identify which locations in the Americas fall within the grackle-suitable ranges in 1970 and in 2018. We would then be able to compare the maps (1970 and 2018) to determine whether the amount of suitable habitat has increased or decreased. If we would be able to find data for these variables before 1970 across the Americas, we would additionally run models using the oldest available data to estimate the range of suitable habitat earlier in the great-tailed grackle range expansion period.

- **Final analysis:** We used the discrimination ability of our SDMs as metrics for how accurately our models predict grackle-suitable habitat and whether one model could be used to predict suitable habitat in both the historic and current time periods for each species. We tested discrimination ability using the 20% of data excluded from the training set of each model. We measured Cohen’s Kappa and AUC for each model. We also used these metrics to quantify model transferability, the ability of a model to perform accurately using datasets independent of the training dataset. Model transferability has been used to measure the consistency of habitat associations over time (Regos, Imbeau, et al., 2018; Torres et al., 2015; Wu, Li, et al., 2016). Low transferability would indicate that the backcast or forecast suitability predictions do not accurately represent the species range and that the relationship between occurrence probability and environmental predictors has changed. We used the 20% excluded from the opposite time period (1970–1979 for the current backcast and 2010–2019 for the historic forecast) model to test the transferability of our models over time. We also compared the geographic extents of suitable habitat based on the historic and current models for both species to determine whether the models agree on the range dynamics for their species (Fig 2). We used the sensitivity-specificity-sum-maximum threshold (Liu, Berry, et al., 2005) to classify suitable habitat. We applied the suitability threshold to the contemporary prediction maps and the backcast/forecast prediction maps to generate predicted suitable habitat ranges in 1979 and 2019. We then mapped changes in habitat suitability classifications to determine the range dynamics predicted by each model.
- **Deviations from the preregistered plan:** We predicted habitat suitability in 1979 and 2019 instead of 1970 and 2018 to line up with the most recent years within our historic and current datasets.

#### Analysis 2: habitat associations:

Does the range of variables that characterize suitable habitat for the great-tailed grackle differ from that of the boat-tailed grackle? We fit species distribution models for both species in 2018 to identify the variables that characterize suitable habitat. We planned to examine the raw distributions of these variables from known grackle occurrence points or extract information on how the predicted probability of grackle presence changes across the ranges for each habitat variable. The habitat variables for each species would be visualized in a figure that shows the ranges of each variable and how much the ranges of the variables overlap between the two species or not.

- **Final analysis:** To determine changes in habitat associations over time, we quantified the importance of each environmental predictor using the Gini index and calculated the partial dependence of each model to the environmental predictors. The Gini index quantifies the classification information gained when a predictor was included in our random forests, with more informative predictors receiving greater values (Strimas-Mackey, Hochachka, et al., 2020). We calculated partial dependence by averaging the predicted habitat suitability across 1000 randomly selected checklists in which one predictor was set to 1 of 25 evenly spaced values across its observed range. We repeated the partial dependence calculation across all 25 values to create a partial dependence curve for every predictor. To compare partial dependence across predictors, we subtracted all partial dependence values by the minimum habitat suitability for each curve to obtain the marginal effect of each predictor.
- **Deviations from the preregistered plan:** We did not compare the distribution of environmental values at observation points. Instead, we used predictor importance and the partial dependence of habitat suitability on each predictor because they are more informative metrics of habitat breadth. Predictor importance and the partial dependence of habitat suitability on each predictor take into account differences in sampling effort across geographic areas and predictor covariation. Comparing the distribution of environmental values at observation points would not have accounted for these confounding effects and would not take full advantage of the information available through our SDMs.

#### Analysis 3: habitat occupancy:

Have the habitats occupied by both species changed over time? We planned to count the number of different land cover categories each species is or was present in during 1970 and 2018. To determine whether land cover influences their distributions, we would calculate how much area in the Americas is in each land cover category, which would then indicate how much habitat is suitable (based solely on land cover) for each species.

- **Final analysis:** We compared the proportion of observations located on each land cover class in addition to the number of different land cover classes that each species was observed on. Changes in the number of land cover classes either species was observed on would indicate that the species occupies novel habitat.

We also performed a niche overlap test using the *ecospat.niche.similarity.test* function within the R package ecospat (Broennimann, Cola, et al., 2023). This function compares the environmental space occupied by the observed points for a species across two different time periods to determine if the differences in the environments that the species are found in across these ranges differ significantly compared to a null space generated by simulations that randomly reassign observations to either time range. We generated the environmental space using a principal component analysis of the environmental predictors found at species occurrence points within both the historic and current time periods. We used the two principal components that explained the largest proportion of variation to create the environmental space because the *ecospat.niche.similarity.test* function is limited to two dimensions. We binned the first two principal components to create a 100×100 grid of environmental predictor values, and we used 100 simulations to create our null expectations. Our two ranges were the historic and current datasets, and we ran the niche overlap test independently for each species. We quantified the niche overlap using Warren’s I (Broennimann, Fitzpatrick, et al., 2012; Warren et al., 2008), a commonly used metric of niche overlap that is calculated using the difference in the occupancy rate of grid cells within the environmental space (frequency of occurrences within each grid cell normalized by the frequency of observations). Lower values of Warren’s I indicate greater differences in the environmental space occupied by the species than expected by chance if the habitat usage for the species is the same across both time ranges. We used Warren’s I instead of the more common Schoerner’s *D* statistic, which Warren’s I is modified from, due to disagreements between these statistics in cases where the ranges compared are drastically different in size (Rödder and Engler, 2011). The historic and current range sizes for the great-tailed grackle differ greatly and could result in the Schoerner’s *D* statistic underestimating niche overlap within the simulations that form the null expectation we compare the observed overlap to. We used direct observations of each species, also known as ordinances, for our niche overlap test instead of the predicted suitability values from our SDMs because ordinance-based tests more accurately quantify niche overlap (Guisan et al., 2014). The niche overlap test excludes areas of niche space that were not sampled within one of the two ranges to avoid non-analogous comparisons.

- **Deviations from the preregistered plan:** We compared species observations from 1970–1979 and 2010–2019 instead of only using observations from 1970 and 2018 to use all available data. We also performed a niche overlap test to compare the observed differences in the environments of the historic and current ranges for each species to a null expectation. Significant differences between the observed habitat occupancy changes and the null expectation indicate that our focal species are occupying different habitats over time.

#### Analysis 4: habitat connectivity:

Has habitat connectivity for both species increased over time? If the connectivity distances are smaller in 2018, this would indicate that habitat connectivity has increased over time. We planned to calculate the least cost path from the northern edge to the nearest suitable habitat patch. To compare the distances between 1970 and 2018, and between the two species, we would run two models where both have the distance as the response variable and a random effect of location to match the location points over time. The explanatory variable for model 1 would be the year (1970, 2018), and for model 2 the species (great-tailed grackle, boat-tailed grackle). If we were be able to find data for these variables before 1970 across the Americas, we would additionally run models using the oldest available data to estimate the range of connected habitat earlier in their range expansion.

- **Final analysis:** We used Circuitscape version 4.0.5 (Anantharaman et al., 2020) to determine whether changes in access to habitat due to connectivity caused by environmental change could explain range shifts in the boat-tailed grackle or the great-tailed grackle. Circuitscape uses electrical circuit theory, treating a landscape as an electrical circuit with different landscape features offering different levels of resistance. We created our resistance surfaces using the results of our current SDMs, which is a common practice when experimental data on species movement through a landscape is not available (Beier et al., 2011; Justen et al., 2021; Miranda et al., 2021). Because we used the current SDMs to create our resistance surfaces, our models tested whether environmental change has connected or isolated areas of suitable habitat given the current realized niche of the species. We converted habitat suitability to resistance using a negative exponential function because this function performs well for avian species (Trainor et al., 2013). Our final resistance surface had values ranging from 1 to 100, with 1 as the minimum resistance value. To calculate connectivity across the entire species range, we used a method that does not require *a priori* selection of habitat patches. This method uses randomly selected points, called nodes, as the locations where current enters and exits the resistance surface (Koen et al., 2014). Connectivity is measured as the current that travels through each cell when moving between these nodes. Current is elevated near the node locations, so we created a buffer surrounding the ranges for each species and selected random points from the perimeter of this buffer for our nodes in Circuitscape (Koen et al., 2014). The elevated connectivity values adjacent to the nodes thus existed outside of the species range, allowing the connectivity values within the species range to remain constant regardless of the location of the randomly selected nodes. The buffer removed the correlation between node location and connectivity values within the checklist ranges, resulting in connectivity values that were only dependent on the resistance map. We used a buffer that was 600 km removed from the edge of the checklist ranges and used 18 randomly selected nodes. We then simulated current between each node using the pairwise function in Circuitscape and used the summed accumulated current as our metric of connectivity. We defined regions within the 75th percentile of the accumulated current values as high connectivity areas because the rank of suitability values, rather than the magnitude of suitability values, are the most transferable feature of SDMs (Guillera-Arroita, Lahoz-Monfort, Elith, et al., 2015). We chose the 75th percentile as our threshold based on (Bonnin et al., 2020).
- **Deviations from the preregistered plan:** We did not calculate the least cost path between habitat patches because we did not have experimental data on species movement nor did we have a priori suitable habitat patches for either species. We used Circuitscape 4.0.5 instead to quantify the accumulated current as a measure of ease of movement through the landscape.

## Results

### Hypothesis 1: Habitat Availability

We compared how habitat availability has changed for the boat-tailed grackle and the great-tailed grackle by predicting habitat suitability across each species range using environmental data from 1979 and 2019 (Analysis 1). We validated these predictions using presence-absence data set aside from the current and historic datasets. If habitat availability was an important factor in determining the range dynamics of either species, then the current models should be sufficient to predict the expected range dynamics, the current and historic models should agree on the locations of suitable habitat, and the current models should be transferable to the historic dataset. Alternatively, if changes in habitat associations or connectivity were important for the species range dynamics, the current and historic models should disagree and be mutually non-transferrable.

**Figure 3 –:**
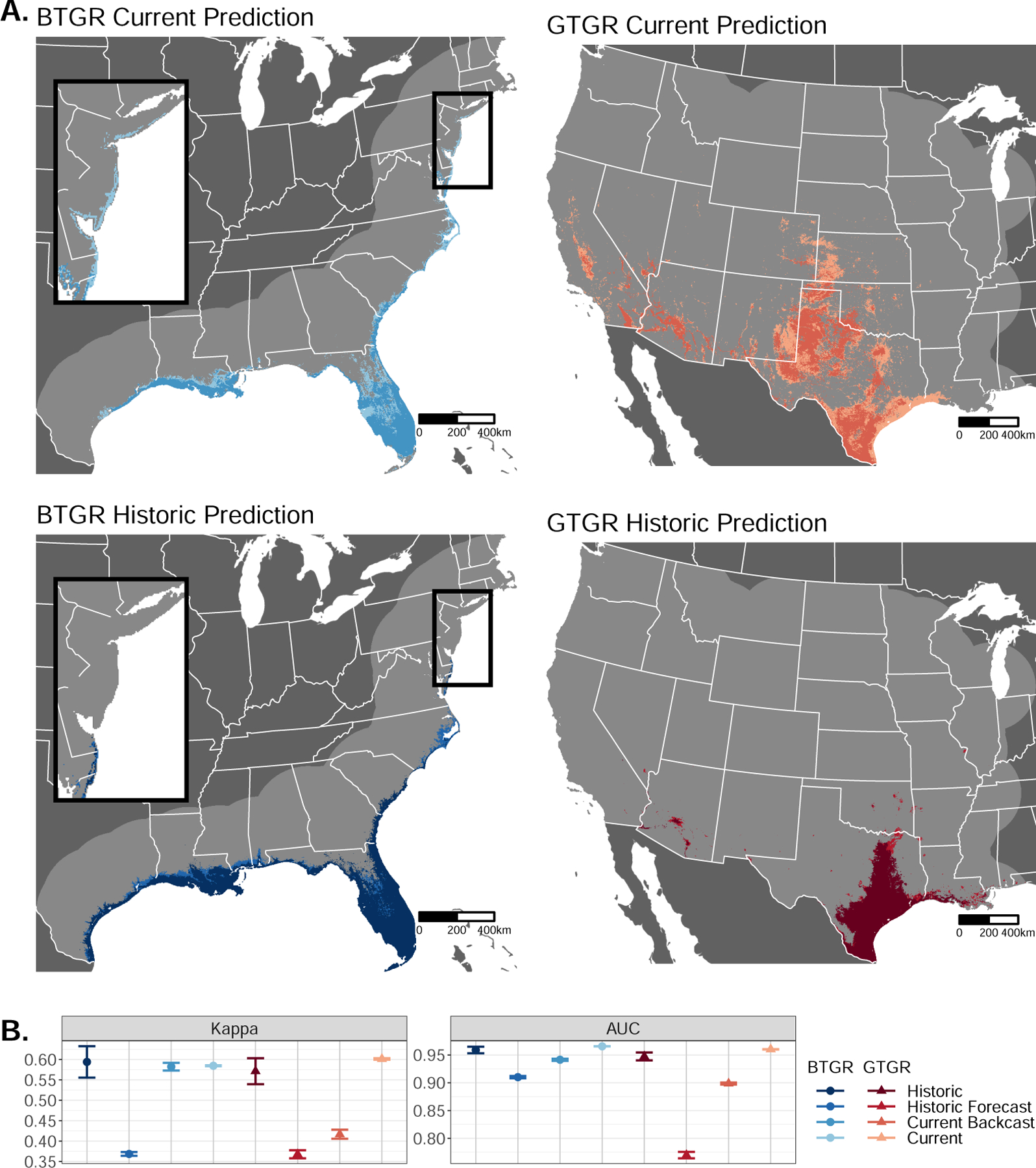
Predicted suitability maps and discrimination ability of SDMs. (A) Maps display areas where predicted suitability is greater than the maximum-sensitivity-specificity thresholds for each model (great-tailed grackle (GTGR) current: 0.4440, boat-tailed grackle (BTGR) current: 0.4780, great-tailed grackle (GTGR) historic: 0.4635, boat-tailed grackle (BTGR) historic: 0.3935). Darker shaded regions are predictions made using the historic environment (historic and current backcast) and lighter regions are predictions made using the current environment (historic forecast and current). The northern edge of the boat-tailed grackle range is expanded in a map insert for clarity. Overall, the areas of lighter color indicate changes in habitat availability from 1979–2019, as predicted by each model. (B) The ability of each model to predict the presence or absence of boat-tailed grackles (blues) or great-tailed grackles (reds) using Cohen’s kappa (agreement between presence or absence classification for model and true presence or absence) and AUC (area under the sensitivity-specificity curve). The models were tested using either test data excluded from the training data set (historic and current predictions) or test data from the opposing temporal period (backcast and forecast predictions). Error bars signify one standard deviation in the values across 10 replicates. The high values of the boat-tailed grackle historic, current backcast, and current, and the great-tailed grackle historic and current models indicate that these models are accurate, while the lower values of the boat-tailed grackle historic forecast and the great-tailed grackle historic forecast and current backcast models indicate that the boat-tailed grackle historic and the great-tailed grackle historic and current models have poor transferability.

Habitat availability for the boat-tailed grackle has remained the same across most of its range according to both the current and historic models, and the current model is highly transferable. The boat-tailed grackle remained restricted to the coasts of the Gulf of Mexico and Atlantic Ocean, but habitat suitability increased within the interior of Florida and on the northern edge of the species range, increasing the total suitable area from 180,406 km^2^ to 199,912 km^2^ in the historic model, and from 111,218 km^2^ to 163,243 km^2^ in the current model (Fig 3A; see Fig S2 for suitability values). The models disagreed on the northern extent of suitable habitat, with the historic model reaching the southern tip of Delaware, while the current model predicted that suitable habitat reached farther north to Long Island. The current model recreated existing species range definitions, including a known break in the species range on the western edge of the Florida panhandle (Post et al., 1996). The current model was also highly transferable, with little difference between the prediction accuracy using the current or historic datasets (∆Kappa = 0, ∆AUC = −0.026, Fig 3B), while the historic model had lower transferability (∆Kappa = −0.226, ∆AUC = −0.049). The accuracy of the current model indicates that environmental change is sufficient to predict changes in habitat suitability, and the low transferability of the historic model could be due to greater geographic bias caused by the smaller sample size (Fig S1). Our models agree with observations that the boat-tailed grackle range has remained largely stable except for an expansion along the northeastern coast of the US and suggest that habitat availability could play a role in the range dynamics of the boat-tailed grackle.

Habitat availability for the great-tailed grackle has expanded, but the current and historical models disagree on the extent and location of this expansion and are mutually non-transferable. The historic model restricted the great-tailed grackle range to 198,175 km^2^ in southern Texas, matching previous reports of the species range in the 1970s (Wehtje, 2003), and predicted minor reductions in range to 181,281 km^2^ (Fig 3A, Fig S2). The current model instead predicted suitable habitat existed in both time periods across the known great-tailed grackle range expansion (Wehtje, 2003) in the central and southwestern US, with further expansions within central California, Colorado, Kansas, and southeastern Texas. Suitable habitat expanded from 322,750 km^2^ in 1979 to 547,694 km^2^ in 2019, however this expansion included areas that were suitable within the historic model. Neither model had high transferability (current: ∆Kappa = −0.184, ∆AUC = −0.061; (historic: ∆Kappa = −0.203, ∆AUC = −0.177, Fig 3B). The disagreement between our models indicates that environmental change alone cannot explain the range expansion of the great-tailed grackle. Each model accurately predicted the species range within its own time period, but failed to predict the known changes in that range. Together, our models predict that the great-tailed grackle range has more than doubled in the past 40 years, but the habitat associations found in one time period are incapable of predicting the changes in occupied habitat over time. These changing habitat associations could indicate that the great-tailed grackle is occupying novel habitat, either because the species can tolerate a wider variety of habitats or has overcome barriers such as dispersal barriers or temporal lag, the time required for populations of a species to establish in previously unoccupied suitable habitat (Essl et al., 2015).

### Hypothesis 2: Habitat Associations

We compared the changes in habitat associations of boat-tailed grackles and great-tailed grackles by measuring the importance of each environmental predictor to the current and historic models for each species and quantifying the marginal effect that changing the value of these predictors had on habitat suitability. Differences in which predictors are most important or how predictors influence habitat suitability describe differences in the realized niches predicted by our models (Analysis 2). We also quantified how frequently each species was observed on different land cover classes between the current and historic datasets to test for changes in the breadth of land cover classes used by either species. Finally, we performed a niche similarity test to determine if the environments occupied by each species in the historic and current time periods are more different from each other than would be expected by chance (Analysis 3). Changes in the environments either species was observed on would indicate that the species has novel habitat associations in the current time period relative to the historic time period.

**Figure 4 –:**
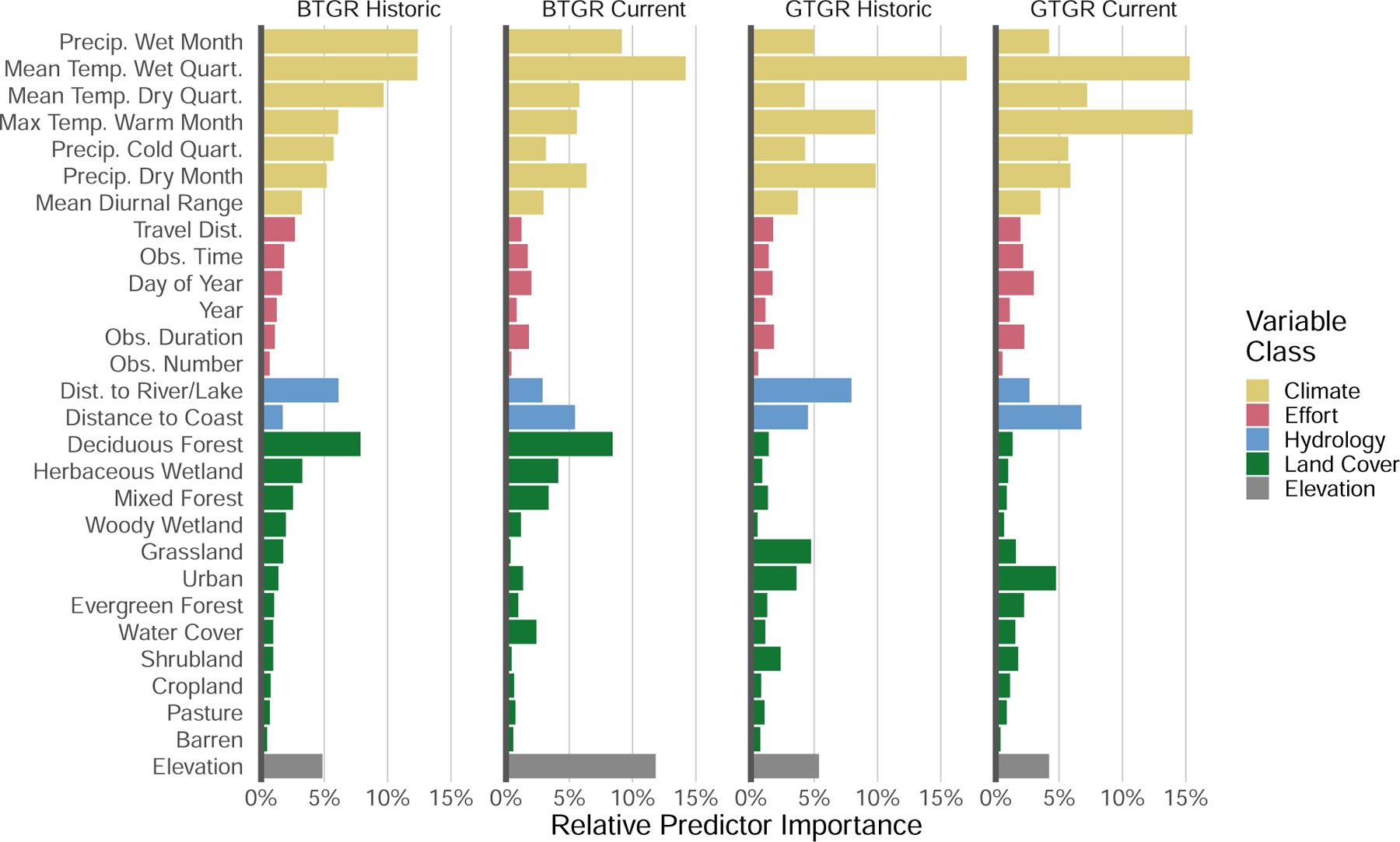
Importance of environmental predictors for the boat-tailed grackle (BTGR) and the great-tailed grackle (GTGR) historic and current species distribution models (SDMs). Relative predictor importance measures how informative the predictors were for classifying presence or absence points within each model (% total GINI index). The predictor colors indicate whether a predictor was a measure of climate (yellow), observer effort (red), distance to water (blue), land cover classification (green), or elevation (gray).

**Figure 5 –:**
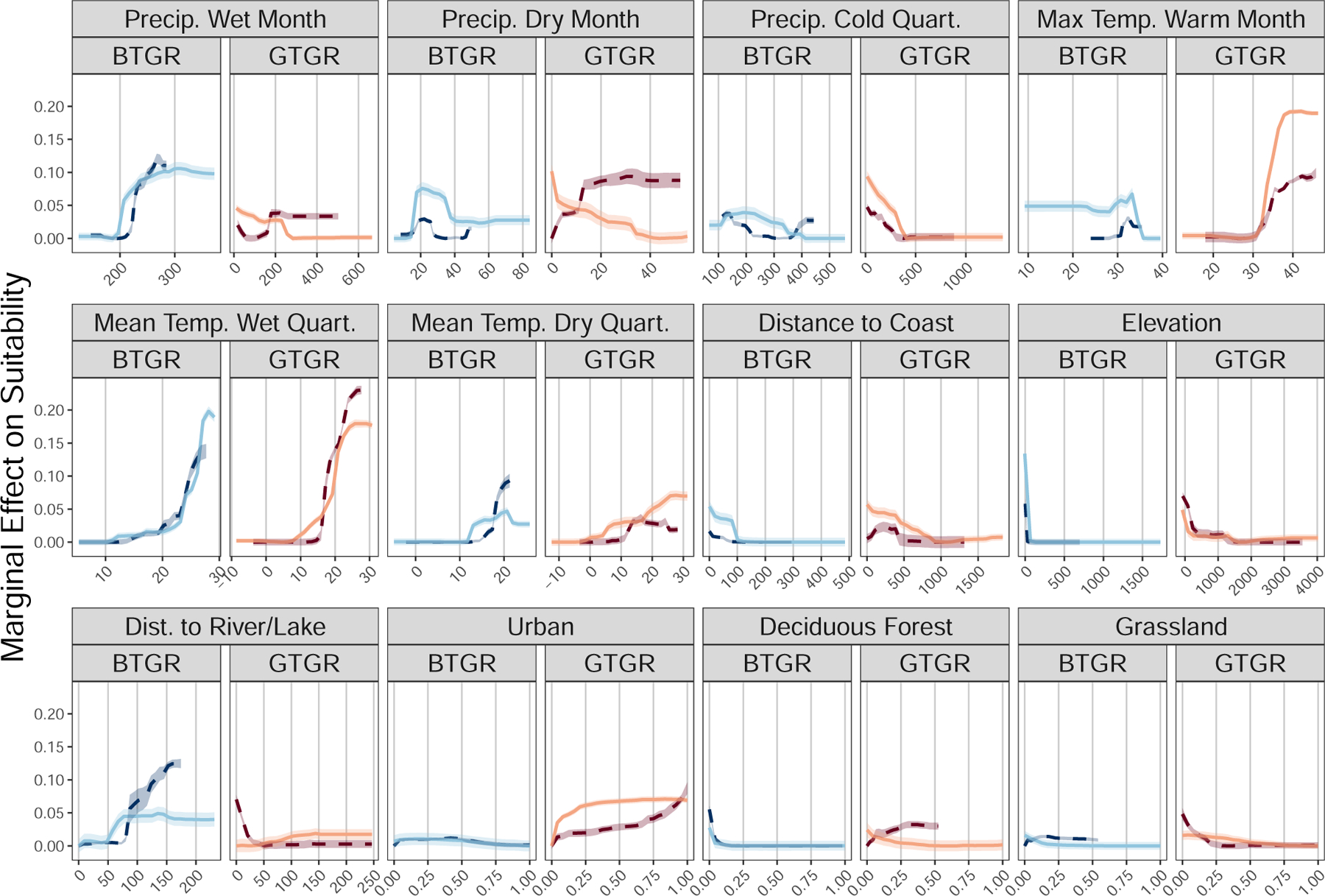
Partial dependence curves for the 12 most important environmental predictors across all boat-tailed grackle (BTGR) and great-tailed grackle (GTGR) models. The curves represent how changing each environmental predictor changes the encounter rate for the modeled species. The historic models are represented by the darker dashed lines and the current models are represented by the lighter solid lines. Shaded regions indicate one standard deviation. The differences between the historic and current models for each species present how realized niches of each species as predicted by our models have changed.

The most important predictors for the current boat-tailed grackle model were mean temperature of the wettest quarter (accounting for 14.2% of the total average GINI index), elevation (11.8%), precipitation of the wettest month (9.1%), and deciduous forest land cover (8.4%; Fig 4). Habitat suitability increased as the mean temperature of the wettest quarter and precipitation of the wettest month increased and was highest when both elevation and deciduous forest land cover were close to zero (Fig 5; see Fig S3 for the full set of partial dependence plots). Our model predicts that the ideal habitats for boat-tailed grackles are warm, low elevation habitats with high precipitation and low forest cover.

The historic model for the boat-tailed grackle disagreed on the importance and effect of only a few predictors, supporting consistent habitat usage in the species. Both the historic and current models placed high importance on the mean temperature in the wettest quarter (12.4%; Fig 4), precipitation of the wettest month (12.4%), and deciduous forest cover (7.9%). However, the historic model prioritized the mean temperature of the driest quarter (9.7%, 5.8% in the current model) and not elevation (4.8%). Among these predictors, only the mean temperature of the driest quarter had a different effect in the historic model than in the current model (Fig 5). Habitat suitability increased as the mean temperature of the driest quarter increased in both models, but the current model predicted that suitability would decrease beyond the observed temperature range of the historic model. Differences between the historic and current models do not support a change in habitat associations of boat-tailed grackles over time.

Boat-tailed grackles were found in every land cover class except deciduous forests and ice/snow in both the historic and current time periods. Boat-tailed grackles were found more often in urban areas in the current time period, and less often in the land cover class that was the second most common in the historic time period: woody wetlands (Fig S4). Boat-tailed grackles were also found less often in croplands, which corresponds with a decrease in croplands across the checklist range. We found no evidence of change in habitat occupancy based on land cover classes for boat-tailed grackles, agreeing with the results of our SDMs. The niche similarity test for the boat-tailed grackle did not find a significant difference in the environmental space occupied by the boat-tailed grackle over time (Warren’s *I* = 0.647; p-value = 0.446, Fig S5B), which further supports the hypothesis that the boat-tailed grackle did not change the environments it occupies between the historic and current time periods.

The most important predictors for the current great-tailed grackle model were maximum temperature of the warmest month (15.5%; Fig 4), mean temperature of the wettest quarter (15.3%), mean temperature in the driest quarter (7.2%), and distance to coasts (6.8%). Habitat suitability increased as the maximum temperature of the warmest month, mean temperature of the wettest quarter, and mean temperature of the driest quarter increased, while suitability was negatively related to the distance to coasts (Fig 5, Fig S3). Our model predicts that the ideal habitats for great-tailed grackles are warm areas not too far from coasts.

The historic model for the great-tailed grackle disagreed on the importance and effect of several predictors, supporting a change in habitat associations. The historic model agreed with the current model on the high importance of the maximum temperature of the warmest month (9.8%, Fig 4) and mean temperature of the wettest quarter (17.0%). However, the historic model prioritized the precipitation in the driest month (9.9% vs. 5.9% in the current model) and the distance to fresh water (7.9% vs. 2.7% in the current model), and not the distance to coasts (4.5%) nor the mean temperature in the driest quarter (4.3%). Habitat suitability increased as precipitation in the driest month increased, while the current model predicted the opposite trend (Fig 5). Habitat suitability was also greatest near fresh water, while the current model predicted little effect of the distance to fresh water. The two models also disagree on which land cover class was most important for great-tailed grackles. Urban cover was most important for the current model (4.8% vs. 3.6% in the historic model), while grassland cover (4.7% vs. 1.5% in the current model) was most important for the historic model. While habitat suitability increased as urban cover increased for both models, the current model reached its maximum suitability by 25% urban cover, while the historic model did not reach similar suitability until almost 100% urban cover. The faster rate of suitability increase in the current model indicates that great-tailed grackles were found across a wide variety of urban habitats, from moderate to highly urbanized areas, while the historic model indicates that great-tailed grackles were preferentially found in highly urbanized habitat. Our models predict that the great-tailed grackle is currently found in more arid habitat with greater variability in urban cover than 40 years ago.

Great-tailed grackles were found in every land cover class except deciduous forests, mixed forests, and ice/snow in the historic sample, and every land cover class except deciduous forests and ice/snow in the current sample. There were more great-tailed grackle observations in the current sample on urban areas, croplands, and grasslands and less observations in water, shrublands, pastures, and evergreen forests (Fig S4). While the most common land cover classes great-tailed grackles were found on had shifted, there was no evidence that great-tailed grackles expanded the breadth of land cover classes they could occupy. These results are consistent with our SDMs, which only found differences in the range of urban habitats that great-tailed grackles occupied. The niche similarity test for the great-tailed grackle found a significant difference in the environmental space occupied by the great-tailed grackle over time (Warren’s *I* = 0.641; p-value = 0.001, Fig S6B). The observed value for Warren’s *I* was lower than the simulated values, further supporting the hypothesis that the great-tailed grackle changed the environments it occupies between the historic and current time periods.

### Hypothesis 3: Connectivity

To determine whether changes in connectivity between habitat patches caused by environmental change could explain the rapid expansion of the great-tailed grackle but not the boat-tailed grackle, we estimated the change in accumulated current across the range of each species between 1979 and 2019 (Analysis 4). Accumulated current summarizes the amount of movement through a cell, thus cells with higher current values are more suitable for movement and increase connectivity. We binned current values into high or low connectivity using the 75th percentile (Bonnin et al., 2020). Most cells within the 75th percentile of current values based on the 1979 resistance surface remained within the 75th percentile for both species. Decreases in the distances between patches of cells with high current between the two time periods would indicate that habitat connectivity has increased.

Connectivity decreased for the boat-tailed grackle along the interior portion of its range (farther from the coasts) in the southern Atlantic states and the southern coast of Texas (Fig S7). However, connectivity increased along the Florida panhandle, the northern coast of North Carolina, and the areas surrounding New York City (New York State, New Jersey, and Connecticut). There were no isolated patches of high connectivity for the boat-tailed grackle, and changes in connectivity did not connect or isolate any habitat patches. Our model does not predict major connectivity changes occurring across the range of the boat-tailed grackle.

Connectivity decreased for the great-tailed grackle within the state of Arizona and along the northern extreme of the cells within the 75th percentile (Oregon, Nevada, Colorado, and Kansas). However, connectivity increased along the eastern extreme (Texas and Oklahoma) and the northern edges in Arizona and New Mexico (Fig S7). Only one region of high connectivity in Montana was isolated from the core of connected cells, and no areas became isolated or connected between 1979 and 2019. Similar to the boat-tailed grackle, our model does not predict major connectivity changes occurring across the range of the great-tailed grackle.

## Discussion

We investigated how changes in habitat availability, habitat breadth, and connectivity relate to differential range dynamics in a sister-species pair. We found that the rapidly-expanding great-tailed grackle has increased the variety of occupied habitats in the past 40 years. The current realized niche of the great-tailed grackle contains more arid climate conditions and is less dependent on bodies of fresh water than in the past realized niche. We did not find evidence for an increase in the connectivity of previously isolated patches of suitable habitat. Overall, our results for the great-tailed grackle are consistent with hypothesis 2, that an expansion in the realized niche of the great-tailed grackle may have contributed to the geographic range expansion of the species (Fig 1). While this expansion might predate the period we investigated, which could be the case if these behavioral traits are part of the inherent repertoire of great-tailed grackles in line with hypothesis 4, the change in the range does not seem to reflect a lag to move into previously unoccupied habitat as the novel habitats the great-tailed grackle now occupies did exist within dispersal distance of the historic range for the species. In contrast, the boat-tailed grackle has remained within the same habitat conditions. Climate change in the northern extreme of the boat-tailed grackle range increased the area of predicted suitable habitat, matching observed expansions of the species in that area. Similar to the great-tailed grackle, we found no changes in connectivity. Accordingly, the range dynamics of the boat-tailed grackle match expectations based on changes in habitat availability, our hypothesis 1 (Fig 1).

Our current boat-tailed grackle model is consistent with past work showing that boat-tailed grackles are highly restricted to coastal areas, and that an expansion into northern coastal areas could be due to climate changes. Boat-tailed grackles rarely occur far from saltwater in the northern portion of their range, but can nest inland across Florida (Post et al., 1996; Selander and Giller, 1961). Our current model recreated this distribution and predicted that elevation and distance to coastline were highly important environmental limitations. The historical model did not recreate the same high suitability within the interior of Florida and had both elevation and distance to coastlines as less important. However, our historic model also had lower transferability and could have reduced accuracy due to a low sample size, which can inflate the impact of geographic bias in samples (Anderson and Gonzalez, 2011; Elith, Kearney, et al., 2010; Guillera-Arroita, Lahoz-Monfort, Elith, et al., 2015; Yates et al., 2018). Our niche similarity test also supports consistent habitat use for the boat-tailed grackle in both time periods. Both SDMs predict increased suitability in the northern portion of the species range, which matches past observations (Selander and Giller, 1961) and general trends observed in several bird species that track their optimal conditions as anthropogenic climate change has altered environments (Chen et al., 2011; Thomas, 2010; Tomiolo and Ward, 2018; Vitousek et al., 1997).

The changes in species range we found in the great-tailed grackle matched those predicted by previous researchers. Selander and Giller, 1961 note that, along the northern range edge, great-tailed grackles have expanded into new arid prairie habitat but were highly restricted to human settlements and farms in these areas. Great-tailed grackles require access to open habitat and standing water across their range (Selander and Giller, 1961), and human land use change and irrigation could meet these needs. Our models did find higher habitat suitability values for the great-tailed grackle close to bodies of freshwater in the historic but not the current time period, suggesting that great-tailed grackles occupy habitats farther from natural open water sources. The differences between the current and historic models were also supported by our niche similarity test, which indicated that great-tailed grackles occupied a significantly different area of environmental space in the current time period relative to the historic time period. The current great-tailed grackle model also predicted higher suitability in areas with more cropland and pasture, but neither land cover class had high predictor importance. Instead, precipitation in the wettest and driest months marked the greatest difference between the current and historic models. Wehtje (2003) proposed that lower nest predation and abundant food in human modified environments could allow the great-tailed grackle to support populations within otherwise suboptimal climate conditions. The great-tailed grackle could use the same land cover classes in both time periods, but current populations have novel or preexisting ways to use human altered environments to expand their realized climatic niche. It is possible that the fundamental niche of the great-tailed grackle has remained the same, while the realized niche has expanded due to anthropogenic environmental change. Our results show that the great-tailed grackle is currently found across a wider variety of broad-scale habitats than 40 years ago. Further work on local-scale habitat use across the range of the great-tailed grackle could explore the causes of the trend we have observed.

It remains unclear why the great-tailed grackle has expanded its realized niche while the boat-tailed grackle has not. Both the boat-tailed grackle and the great-tailed grackle are highly adaptable species with similar foraging habits. Human-associated species like boat-tailed grackles and great-tailed grackles that use urban habitats are typically more behaviorally flexible and better suited to use new environments than other species (Sol, Duncan, et al., 2005; Sol, Lapiedra, et al., 2013; Sol, Timmermans, et al., 2002; Wong and Candolin, 2015). There could be meaningful differences in the degree of flexibility between these species or other factors that limit the ability of the boat-tailed grackle to expand to new habitats. The greater nest-site specificity of the boat-tailed grackle could be a limiting factor, though nest-site plasticity does exist in the species (Post et al., 1996). Further studies are needed to compare ecologically relevant differences in flexibility, exploration, dispersal, and reproductive behaviors between these two species.

Our results demonstrate vastly different niche dynamics within closely related species and illustrate the divergent responses species can have to anthropogenic change. The distinct niche dynamic of each species represents opposing responses to anthropogenic change: the boat-tailed grackle has shifted its range in response to climate change, while the rapidly expanding great-tailed grackle has acclimated to new climates possibly due to human land-use change. Species with similar responses to the boat-tailed grackle could be more vulnerable to future climate change (Thomas, 2010), while the great-tailed grackle parallels rapidly expanding introduced species, despite being native to North America (Peer, 2011). The expansion habitats used by the great-tailed grackle also confounds our ability to project how the species range will change in the future, and could have implications for a projected expansion in the common grackle (*Quiscalus quiscalus*) (Capainolo et al., 2021). Evidence of bird species not following predicted range shifts in response to climate change is building, with many species becoming decoupled from previously identified climatic niches (Viana and Chase, 2022). Species appear to shift their ranges in ways that do not directly track the rapid changes in climate (Currie and Venne, 2017), potentially because the local climate shapes niches indirectly by leading to habitat changes that often can take many years to fully manifest (Neate-Clegg et al., 2020). Identifying the mechanism of range dynamics in both grackle species expands the knowledge of the complex and changing factors that shape species ranges globally.

The high accuracy of our SDMs when cross validated on their own datasets and the transferability of the current boat-tailed grackle model support the use of SDMs as tools to study how species ranges change over time. While improving model transferability remains a challenge for SDMs (Vaughan and Ormerod, 2005; Yates et al., 2018), using a combination of climate and land use data can improve model accuracy and transferability in some situations (Elith and Graham, 2009; Regos, Gagne, et al., 2019). Our results also stress the importance of testing model transferability before assuming niche conservatism for all species. While the niches of species commonly remain consistent (Liu, Wolter, et al., 2020), assuming species will retain their niche through time can limit the usefulness of SDMs. When model transferability is tested, SDMs become a more effective tool for studying species ranges to both understand fundamental questions in ecology and evolution and set conservation priorities in the face of ongoing anthropogenic changes (Chen et al., 2011; Elith, Kearney, et al., 2010; Grenouillet and Comte, 2014; Sofaer et al., 2018).

SDMs are accompanied by several limitations that are important to consider. SDMs are correlative in nature and are susceptible to biases in sample and parameter selection (Regos, Gagne, et al., 2019; Sofaer et al., 2018). Here, we used geographic undersampling and a balanced random forest design to reduce the impact of sampling bias and selected both climate and land cover parameters to include biologically relevant variables, but other potentially causative variables could remain. We note that our results capture correlations between species occurrence and environmental factors, and thus cannot determine a causal link between where either species is found and the environment. Habitat occupancy change could occur independently of environmental change, such as if all suitable sites were not yet occupied due to temporal lag. Increased occupancy as the species reaches already suitable sites would correlate with further environmental change and be captured by our species distribution models. Our models similarly cannot distinguish lagged responses to environmental trends that pre-date our dataset from responses to within-dataset trends. The temporal limits of our study could influence our results as the species ranges could react to changes beyond the scales we investigated. Environmental change that occurred before 1970 could have influenced the observed ranges of the species during 1970–1979 due to temporal lag in the species occupying areas within their fundamental niches. Because our models were trained on species occurrences, the niches described by our model depend on a combination of environmental factors that are physiologically or behaviorally favored by the species (the fundamental niche for the species), dispersal behavior and limitations, and biotic factors that influence where the two species will occur (Soberón and Nakamura, 2009). We included a broad set of climatic, land use, topographic, and hydrologic factors within our SDMs to capture the environmental factors that could influence occurrence, but these factors may be incomplete, or may be too coarse to capture local scale habitat use. Our connectivity analysis investigated whether environmental change could influence the dispersal limitations for either species, but assumed that dispersal ability and habitat use remained constant over time. Further work is needed to investigate variation in dispersal behavior within the great-tailed grackle and boat-tailed grackle to determine the possible influence of dispersal behavior in the range dynamics for both species (see Q1 and Q2 of Logan et al., 2020 for project proposals). Recent work promotes the inclusion of biotic factors in SDMs such as pathogen, predator, or competitor species because interspecific dynamics can play a major role in determining species ranges (Gaston, 2003; Paquette and Hargreaves, 2021; Stephan et al., 2021). Determining the relevant biotic factors for each species remains challenging, but future work could investigate how the presence of nest predators such as the fish crow (*Corvus ossifragus*), which overlaps in range with boat-tailed grackles but not great-tailed grackles (Post et al., 1996), could also prevent the boat-tailed grackle from expanding its range.

In conclusion, this investigation found that across the range expansion of the great-tailed grackle, the species now occupies a wider variety of habitats than 40 years ago, while the boat-tailed grackle is found within the same habitats over time, even as environments have changed. Despite the many similarities between these two species, they occupy distinct niches and appear to have divergent responses to anthropogenic change. While the boat-tailed grackle range currently conforms to climate change, the great-tailed grackle has expanded across new human-altered environments. The potential causes for the observed widening of habitat use in the great-tailed grackle, but not the boat-tailed grackle, demand further investigation of the ecology, gene flow, and behavior of both species that could have created such different range dynamics. We encourage others to also consider behavior when attempting to understand what limits species ranges (e.g., Greggor et al., 2016). Here we have detailed how environmental and habitat use change can play important roles in range expansions and range stability, and future work will elucidate the factors shaping species ranges in our rapidly changing world.

## Supplementary Material

1 **Supplementary Figure 1** – Map of observation locations for boat-tailed grackles (BTGR) or greattailed grackles (GTGR) from historic (1970–1979) and current (2010–2019) eBird records. These locations are filtered for record quality
**Supplementary Figure 2** – Predicted habitat suitability using random forest models for boat-tailed grackles (BTGR) and great-tailed grackles (GTGR). Brighter colors indicate higher habitat suitability. The presented results are the average of the 10 replicates.
**Supplementary Figure 3** – Partial dependence curves for environmental predictors across all models (boat-tailed grackle: BTGR; great-tailed grackle: GTGR). The curves represent how changing each environmental predictor changes the encounter rate for the modeled species. The historic models are represented by the darker dashed lines and the current models are represented by the lighter solid lines. Shaded regions indicate one standard deviation. The differences between the historic and current models for each species present how the species niche has changed based on our models.
**Supplementary Figure 4** – Land cover classes with observations of boat-tailed grackles (BTGR) and great-tailed grackles (GTGR) in 1970–1979 and 2010–2019 compared to the change in percent land cover area between each year range. The proportion of land cover measures what percent of observations for each species were located on each land cover class in the corresponding time frame. Both species were found more often in urban environments in the current time period, which also corresponds with a slight increase in the urban background area. Both species were also found less often in their previously second most common land cover type (woody wetland for boat-tailed grackles and shrubland for great-tailed grackles).
**Supplementary Figure 5** – Results of the niche similarity test between the historic (1970–1979) and current (2010–2019) time periods for the boat-tailed grackle. (A) Species occurrence points plotted along the first two principal component (PC) axes used for the niche similarity test. The percent variance captured by each principal component is presented in the axis label. The black lines expanding from the origin indicate the rotation values for the environmental predictors along the two principal components. The current time period observations were randomly subsampled to 1000 points for visual clarity. (B) Values of Warren’s *I* from the niche similarity test based on the observed data (solid line) and 100 simulations (histogram). Higher values of Warren’s *I* indicate greater niche similarity. The p-value presented for the observed value is based on the null hypothesis that the observed value presents equal or greater niche similarity than the simulations.
**Supplementary Figure 6** – Results of the niche similarity test between the historic (1970–1979) and current (2010–2019) time periods for the great-tailed grackle. (A) Species occurrence points plotted along the first two principal component (PC) axes used for the niche similarity test. The percent variance captured by each principal component is presented in the axis label. The black lines expanding from the origin indicate the rotation values for the environmental predictors along the two principal components. The current time period observations were randomly subsampled to 1000 points for visual clarity. (B) Values of Warren’s *I* from the niche similarity test based on the observed data (solid line) and 100 simulations (histogram). Higher values of Warren’s *I* indicate greater niche similarity. The p-value presented for the observed value is based on the null hypothesis that the observed value presents equal or greater niche similarity than the simulations.
**Supplementary Figure 7** – Change in connectivity between 1979 and 2019 measured as change in accumulated current for boat-tailed grackles (BTGR) and great-tailed grackles (GTGR). Current values were divided into high and low categories based on whether the values were above or below the 75th percentile of current values for each map. Colors indicate whether the current values remained low between the two time steps (gray), went from high to low (magenta), went from low to high (blue), or remained high (green). The darker gray color indicates areas outside the range where checklists were selected for each species, and were excluded from the connectivity analysis. The regions that have remained highly connected are continuous for both species, which indicates that changes in connectivity are not responsible for range changes in either species..

## Data Availability

All data and code used in this study are available at the associated KNB repository (https://knb.ecoinformatics.org/view/doi:10.5063/F11G0JR2; Summers et al., 2023)
