# Supplementary material for: The role of climate change and niche shifts in divergent range dynamics of a sister-species pair
Source: Peer Community J. Author manuscript; Available in PMC 2023 Jul 7. (PMC10328137; doi:10.24072/pcjournal.248)
Supplement: 1 — Supplementary Figure 1 – Map of observation locations for boat-tailed grackles (BTGR) or greattailed grackles (GTGR) from historic (1970–1979) and current (2010–2019) eBird records. These locations are filtered for record quality Supplementary Figure 2 – Predicted habitat suitability using random forest models for boat-tailed grackles (BTGR) and great-tailed grackles (GTGR). Brighter colors indicate higher habitat suitability. The presented results are the average of the 10 replicates. Supplementary Figure 3 – Partial dependence curves for environmental predictors across all models (boat-tailed grackle: BTGR; great-tailed grackle: GTGR). The curves represent how changing each environmental predictor changes the encounter rate for the modeled species. The historic models are represented by the darker dashed lines and the current models are represented by the lighter solid lines. Shaded regions indicate one standard deviation. The differences between the historic and current models for each species present how the species niche has changed based on our models. Supplementary Figure 4 – Land cover classes with observations of boat-tailed grackles (BTGR) and great-tailed grackles (GTGR) in 1970–1979 and 2010–2019 compared to the change in percent land cover area between each year range. The proportion of land cover measures what percent of observations for each species were located on each land cover class in the corresponding time frame. Both species were found more often in urban environments in the current time period, which also corresponds with a slight increase in the urban background area. Both species were also found less often in their previously second most common land cover type (woody wetland for boat-tailed grackles and shrubland for great-tailed grackles). Supplementary Figure 5 – Results of the niche similarity test between the historic (1970–1979) and current (2010–2019) time periods for the boat-tailed grackle. (A) Species occurrence points plotted along [file NIHMS1889534-supplement-1.pdf]

Appendix

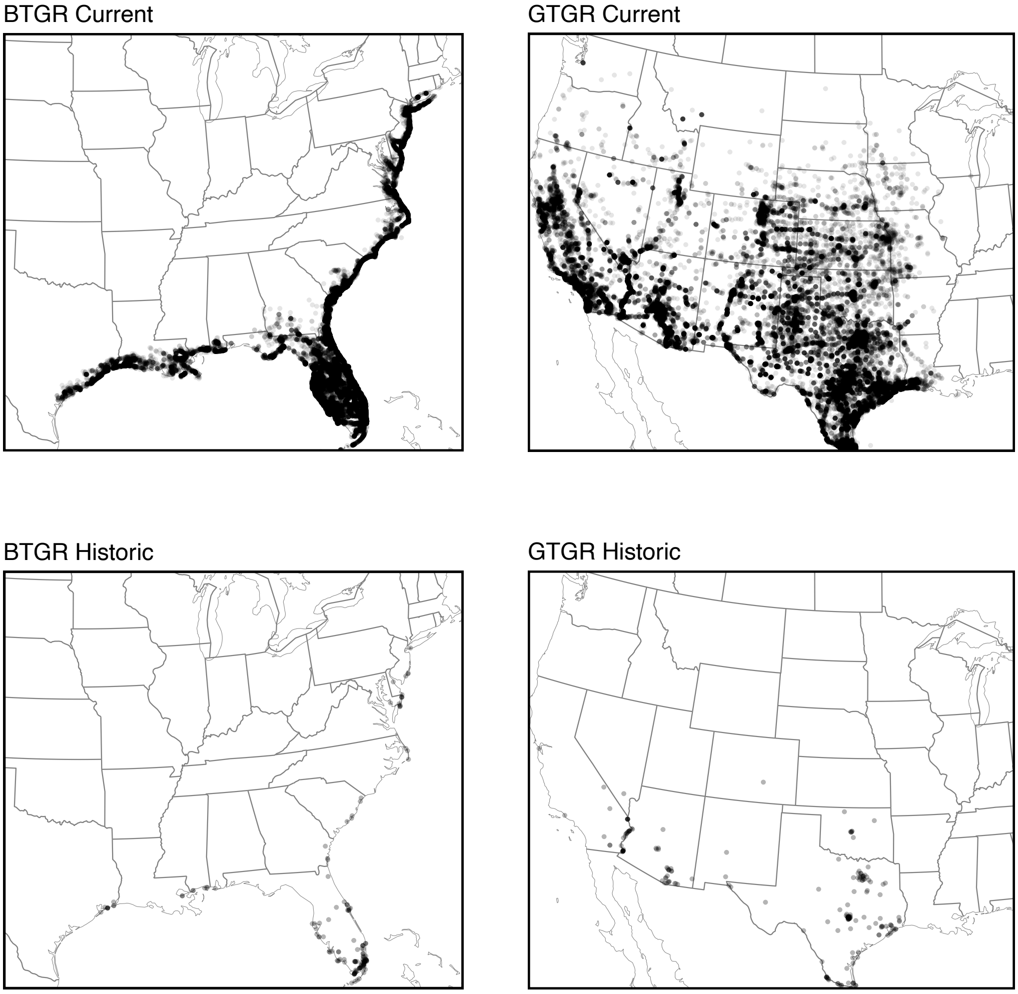

Supplementary Figure 1 – Map of observation locations for boat-tailed grackles (BTGR) or great-tailed grackles (GTGR) from historic (1970-1979) and current (2010-2019) eBird records. These locations are filtered for record quality

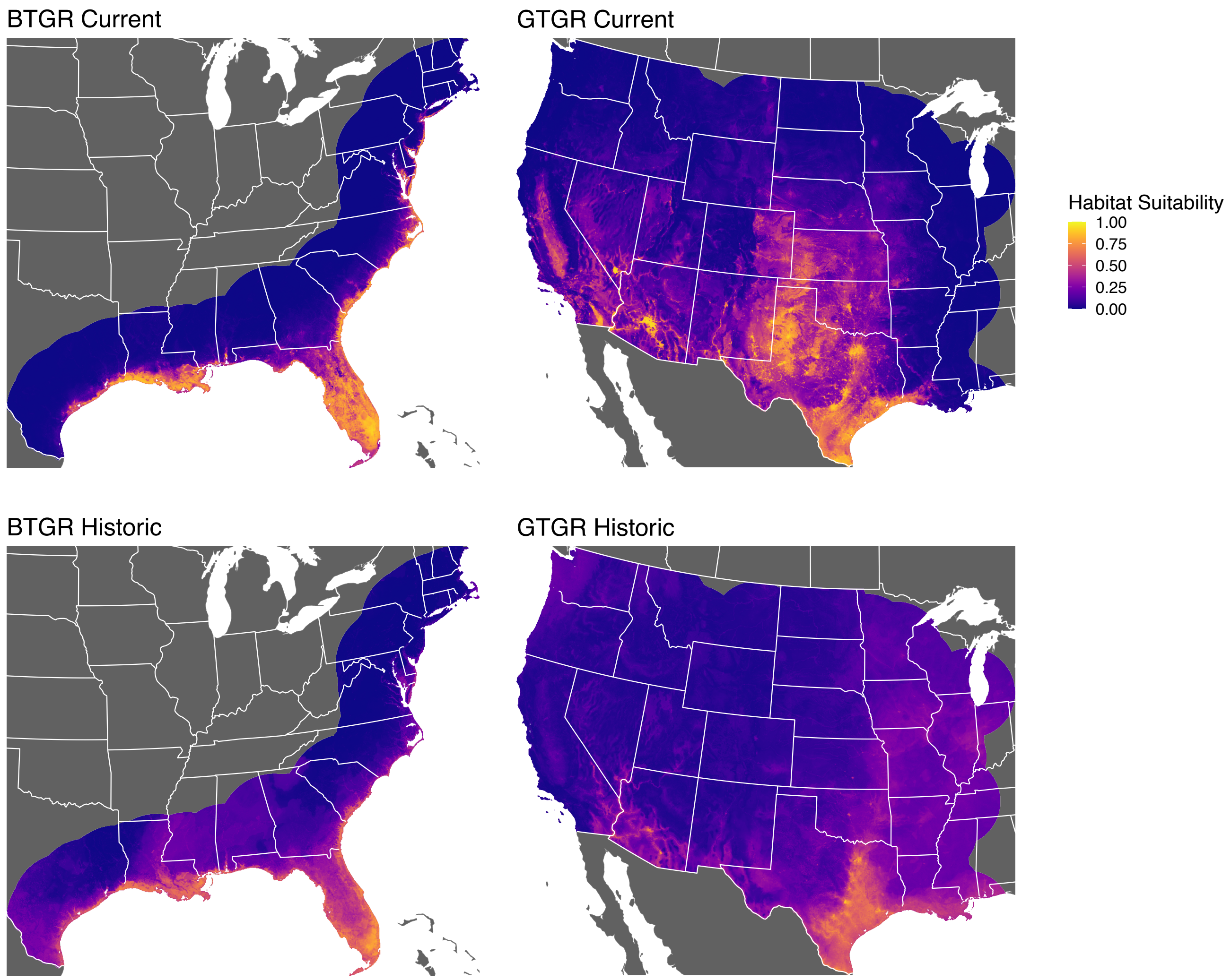

Supplementary Figure 2 – Predicted habitat suitability using random forest models for boat-tailed grackles (BTGR) and great-tailed grackles (GTGR). Brighter colors indicate higher habitat suitability. The presented results are the average of the 10 replicates.

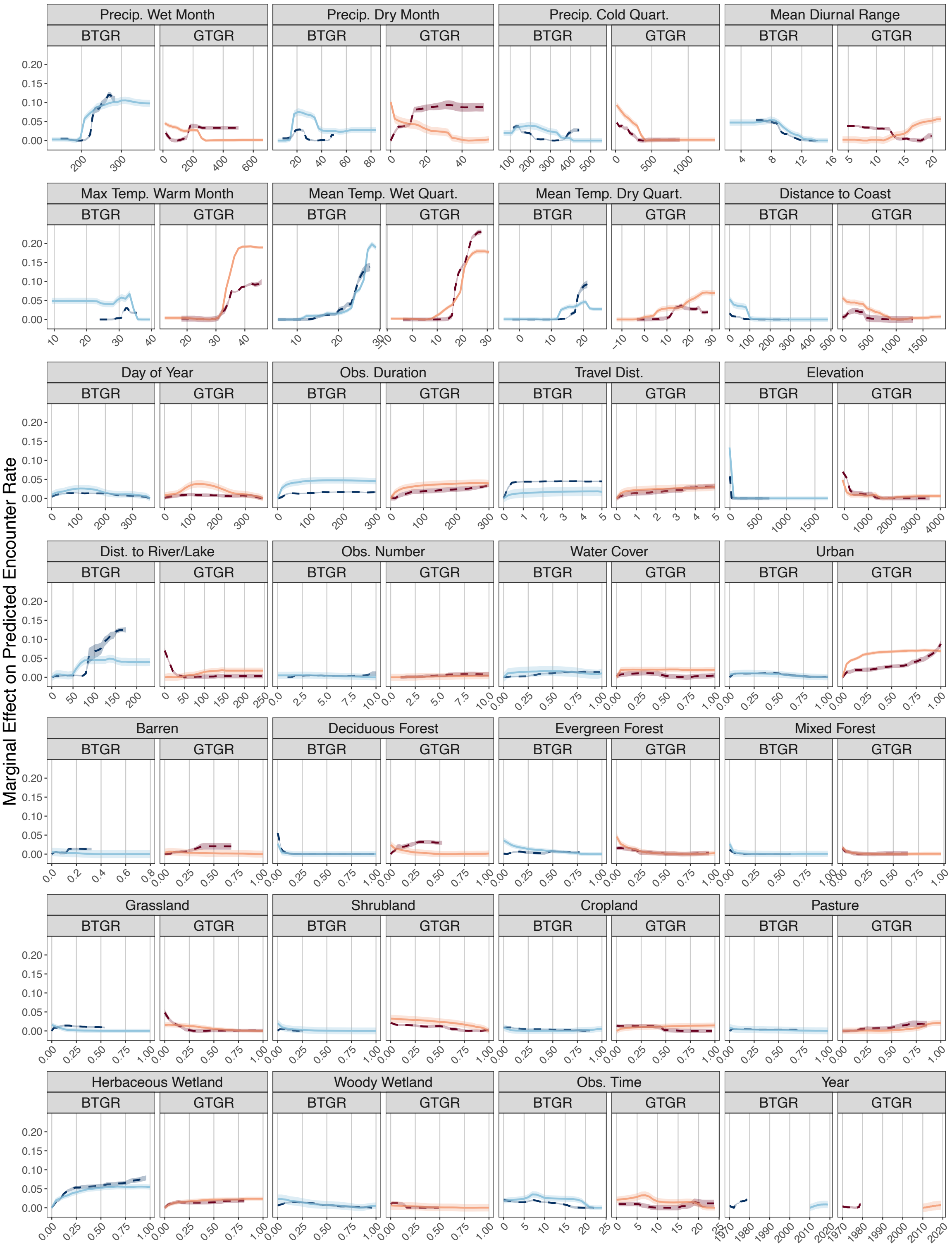

Supplementary Figure 3 – Partial dependence curves for environmental predictors across all models (boat-tailed grackle: BTGR; great-tailed grackle: GTGR). The curves represent how changing each environmental predictor changes the encounter rate for the modeled species. The historic models are represented by the darker dashed lines and the current models are represented by the lighter solid lines. Shaded regions indicate one standard deviation. The differences between the historic and current models for each species present how the species niche has changed based on our models.

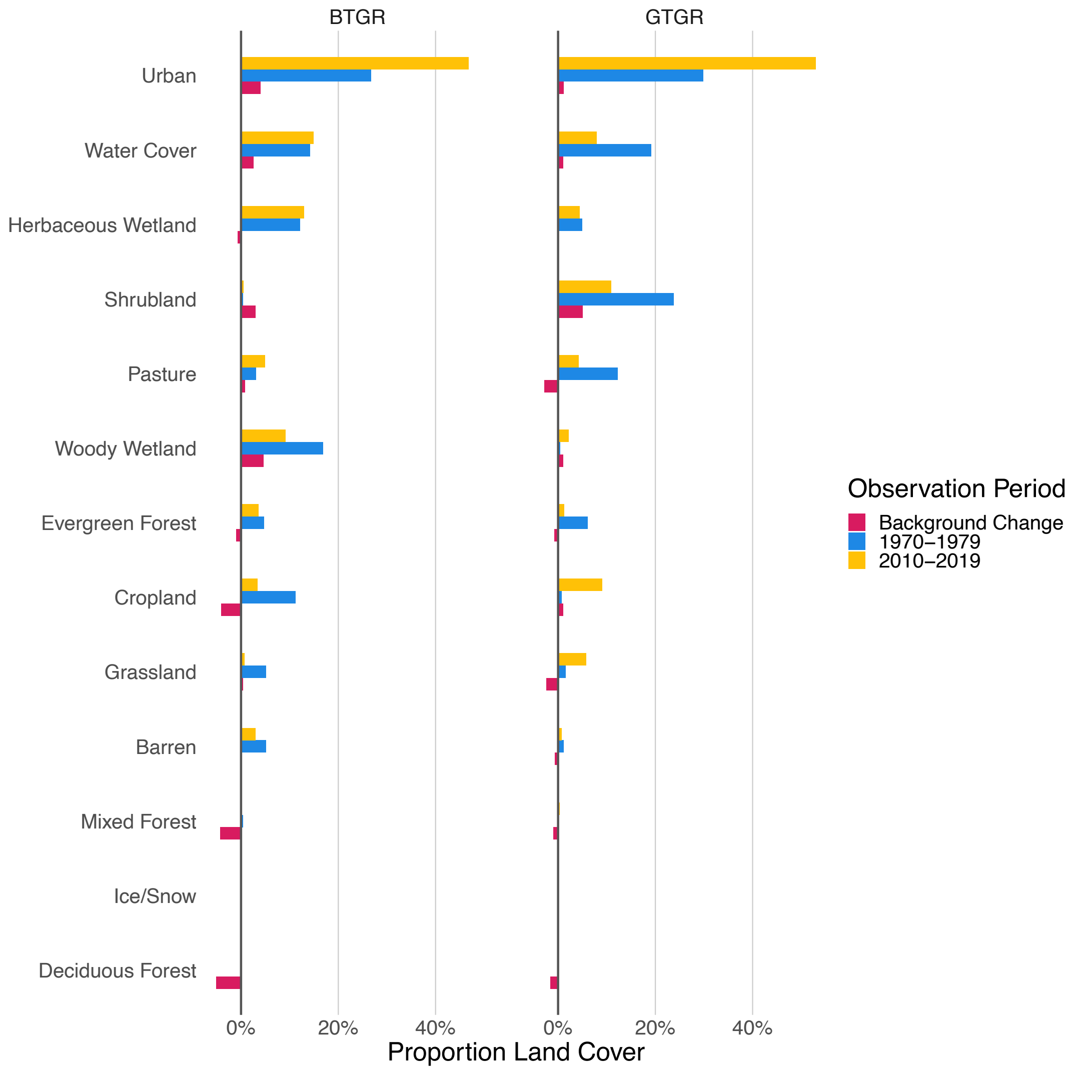

Supplementary Figure 4 – Land cover classes with observations of boat-tailed grackles (BTGR) and great-tailed grackles (GTGR) in 1970-1979 and 2010-2019 compared to the change in percent land cover area between each year range. The proportion of land cover measures what percent of observations for each species were located on each land cover class in the corresponding time frame. Both species were found more often in urban environments in the current time period, which also corresponds with a slight increase in the urban background area. Both species were also found less often in their previously second most common land cover type (woody wetland for boat-tailed grackles and shrubland for great-tailed grackles).

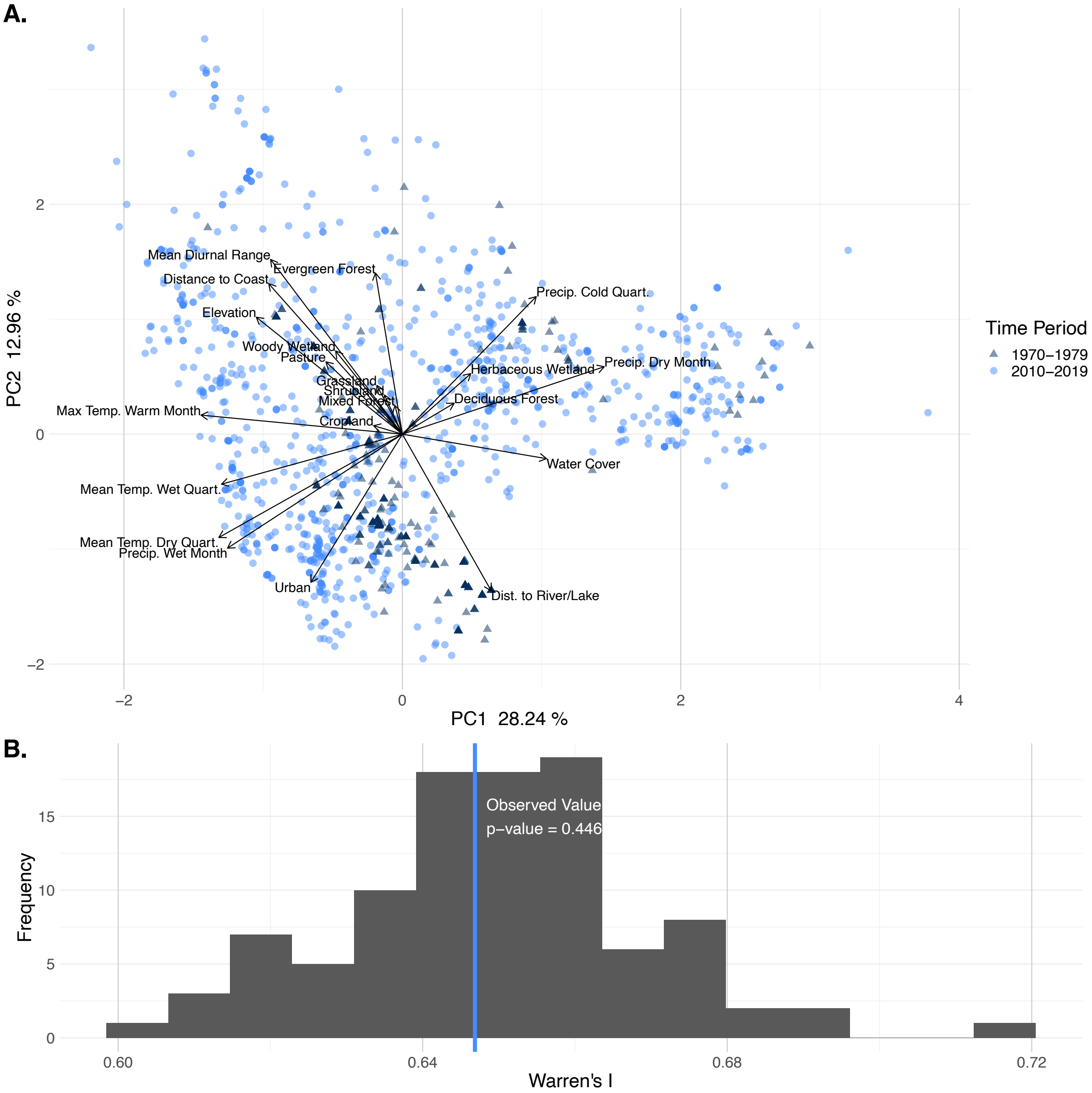

Supplementary Figure 5 – Results of the niche similarity test between the historic (1970-1979) and current (2010-2019) time periods for the boat-tailed grackle. (A) Species occurrence points plotted along the first two principal component (PC) axes used for the niche similarity test. The percent variance captured by each principal component is presented in the axis label. The black lines expanding from the origin indicate the rotation values for the environmental predictors along the two principal components. The current time period observations were randomly subsampled to 1000 points for visual clarity. (B) Values of Warren's *I* from the niche similarity test based on the observed data (solid line) and 100 simulations (histogram). Higher values of Warren's *I* indicate greater niche similarity. The p-value presented for the observed value is based on the null hypothesis that the observed value presents equal or greater niche similarity than the simulations.

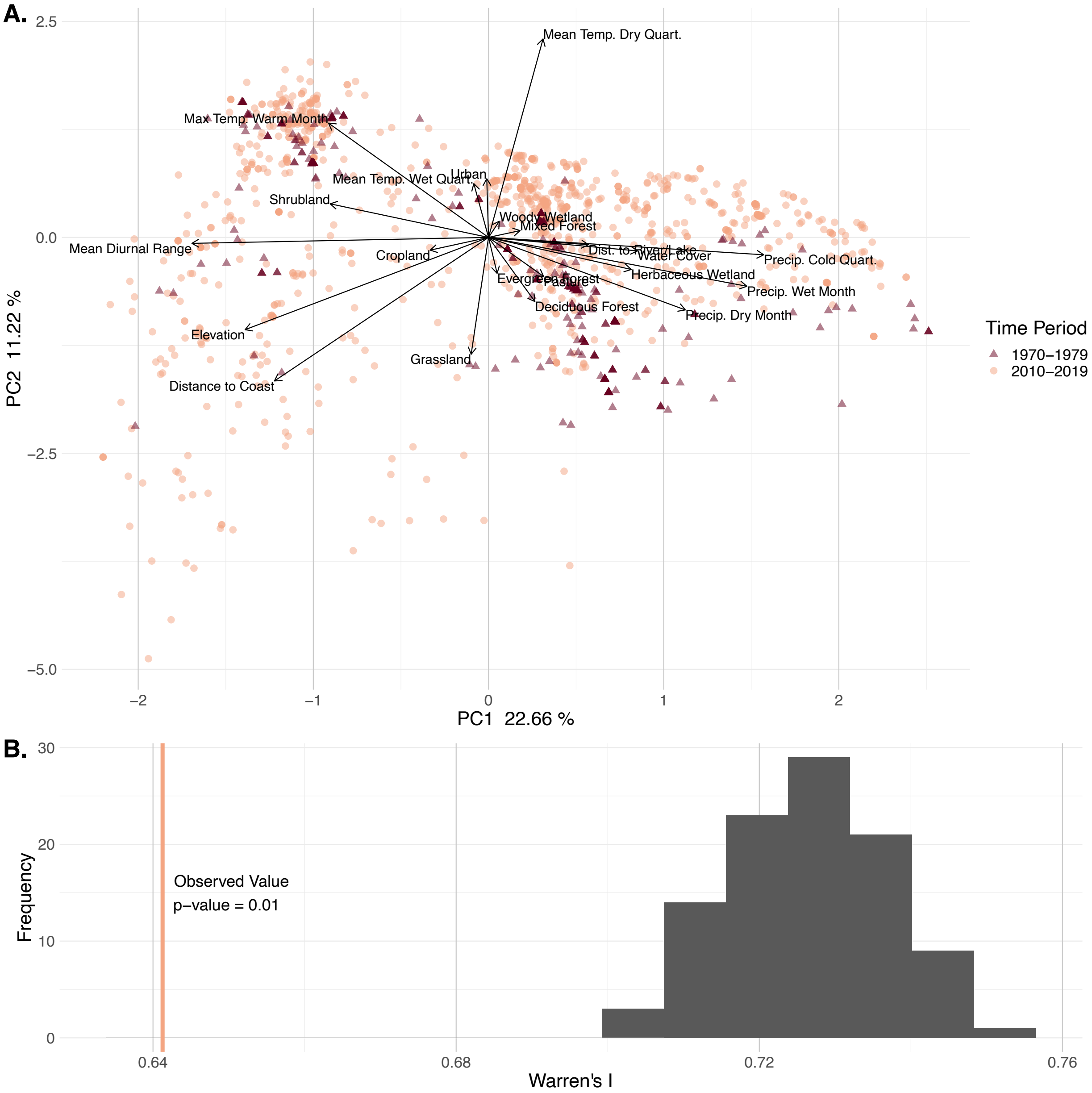

Supplementary Figure 6 – Results of the niche similarity test between the historic (1970-1979) and current (2010-2019) time periods for the great-tailed grackle. (A) Species occurrence points plotted along the first two principal component (PC) axes used for the niche similarity test. The percent variance captured by each principal component is presented in the axis label. The black lines expanding from the origin indicate the rotation values for the environmental predictors along the two principal components. The current time period observations were randomly subsampled to 1000 points for visual clarity. (B) Values of Warren's *I* from the niche similarity test based on the observed data (solid line) and 100 simulations (histogram). Higher values of Warren's *I* indicate greater niche similarity. The p-value presented for the observed value is based on the null hypothesis that the observed value presents equal or greater niche similarity than the simulations.

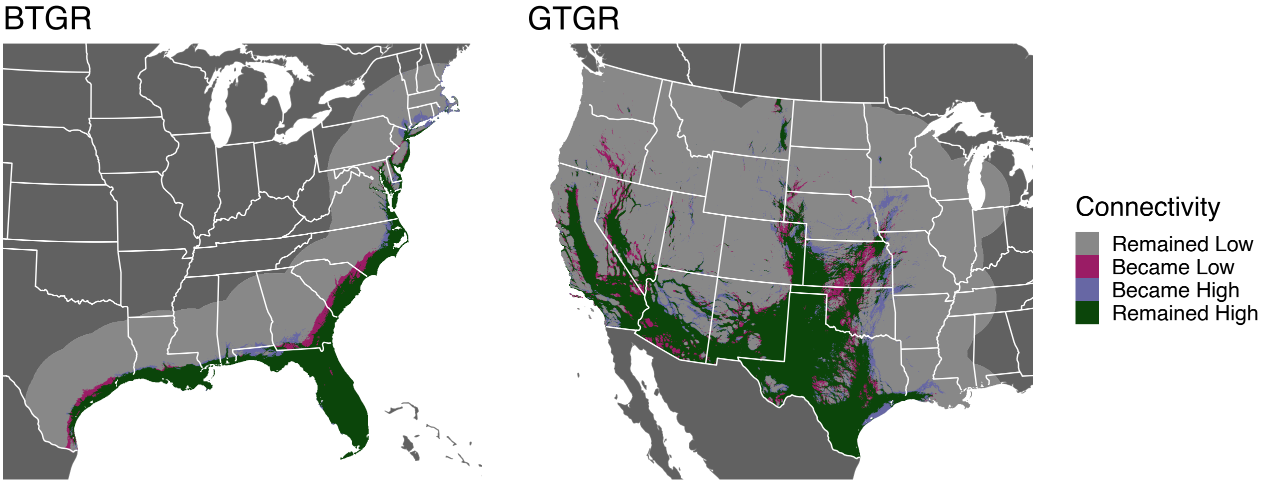

Supplementary Figure 7 – Change in connectivity between 1979 and 2019 measured as change in accumulated current for boat-tailed grackles (BTGR) and great-tailed grackles (GTGR). Current values were divided into high and low categories based on whether the values were above or below the 75th percentile of current values for each map. Colors indicate whether the current values remained low between the two time steps (gray), went from high to low (magenta), went from low to high (blue), or remained high (green). The darker gray color indicates areas outside the range where checklists were selected for each species, and were excluded from the connectivity analysis. The regions that have remained highly connected are continuous for both species, which indicates that changes in connectivity are not responsible for range changes in either species..
